# Superior temporal gyrus and cerebellar loops predict nonsuicidal self-injury in major depressive disorder patients by multimodal neuroimaging

**DOI:** 10.1038/s41398-022-02235-y

**Published:** 2022-11-10

**Authors:** Lijun Kang, Wei Wang, Nan Zhang, Zhaowen Nie, Qian Gong, Lihua Yao, Ning Tu, Hongyan Feng, Xiaofen Zong, Hanping Bai, Gaohua Wang, Lihong Bu, Fei Wang, Zhongchun Liu

**Affiliations:** 1 Department of Psychiatry, Renmin Hospital of Wuhan University, Wuhan, China; 2 PET/CT/MRI and Molecular Imaging Center, Renmin Hospital of Wuhan University, Wuhan, China; 3 Early Intervention Unit, Department of Psychiatry, Affiliated Nanjing Brain Hospital, Nanjing Medical University, Nanjing, China; 4 Functional Brain Imaging Institute of Nanjing Medical University, Nanjing, China; 5 Taikang Center for Life and Medical Sciences, Wuhan University, Wuhan, China

**Keywords:** Human behaviour, Scientific community

## Abstract

In major depressive disorder (MDD) patients, nonsuicidal self-injury (NSSI) is a common comorbidity, and it is important to clarify the underlying neurobiology. Here, we investigated the association of NSSI with brain function and structure in MDD patients. A total of 260 MDD patients and 132 healthy controls (HCs) underwent resting-state functional magnetic resonance imaging and three-dimensional T1-weighted structural scans. NSSI behaviour was assessed through interviews. Voxel-based morphometry analysis (VBM), regional homogeneity analysis (ReHo), functional connectome topology properties and network-based statistics were used to detect the differences in neuroimaging characteristics. Finally, the random forest method was used to evaluate whether these factors could predict NSSI in MDD. Compared with HCs, MDD patients with a history of NSSI showed significant right putamen grey matter volume (GMV), right superior orbital frontal cortex ReHo, left pallidum degree centrality, and putamen-centre function network differences. Compared to MDD subjects without NSSI, those with past NSSI showed significant right superior temporal gyrus (STG) GMV, right lingual gyrus ReHo, sigma and global efficiency, and cerebellum-centre function network differences. The right STG GMV and cerebellum-centre function network were more important than other factors in predicting NSSI behaviour in MDD. MDD patients with a history of NSSI have dysregulated spontaneous brain activity and structure in regions related to emotions, pain regulation, and the somatosensory system. Importantly, right STG GMV and cerebellar loops may play important roles in NSSI in MDD patients.

## Introduction

Major depressive disorder (MDD) is considered the most prevalent mental disease worldwide and has devastating social and personal consequences, affecting more than 350 million people worldwide each year [1]. Although significant progress has been made in understanding MDD, much is unknown about the neuropathology of the disease and its pathophysiology with varying clinical characterizations [2, 3]. Nonsuicidal self-injury (NSSI) is a deliberately damaging behaviour without suicidal intent [4]. In MDD, NSSI history is associated with more severe clinical symptoms, such as the severity of depression symptoms, treatment response, and risk of recurrence [5]. Importantly, a history of NSSI correlates with suicidal behaviour and suicide attempts and increased morbidity and mortality, further underlining the need for research on why and how people experience NSSI [6]. Our previous research and other groups’ studies have found that approximately one-third of MDD patients have a history of NSSI and high comorbidities, as well as many common risk factors, such as family and cultural background, personality characteristics, childhood abuse, cognitive distortion, and low self-esteem [7–9]. Therefore, exploring the neurobiological signature of MDD from NSSI history perspectives may have the potential to transform current conceptualizations of the disease and sharpen the search for treatment targets.

Research on the neurobiology of NSSI has only recently emerged, but initial reports suggest that NSSI may indeed exhibit activation changes, including increased anterior cingulate cortex (ACC) [8, 10], increased orbitofrontal cortex (OFC) activation, decreased dorsolateral prefrontal cortex and decreased amygdala activation in task-based MRI[10]. Changes in the function of these areas are inversely correlated with emotional reactivity and self-reported impulsivity[10] A recent review showed that NSSI exhibited reduced ACC volume and frontolimbic alterations and blunted striatal activation related to top-down and bottom-up neural alterations [11]. In different populations, the changes in brain activity involved in the occurrence of NSSI are different. Multimodal neuroimaging of adolescents with NSSI shows enhanced emotional reactivity that is associated with the anterior insula response [12] and reduced bilateral amygdala activation during reward anticipation [13]. In self-injuring adolescent girls, whole-brain analyses revealed reduced insular cortex bilaterally and right inferior frontal gyrus grey matter volumes in task based MRI [14]. Regional grey matter volumes (GMVs) of the insula and ACC volume showed significant associations with past suicide attempts [15]. There has been only one imaging NSSI study of MDD patients in resting state, and the results show that depressed adolescents with NSSI have default mode networks and insula-salience networks associated with difficulties in self-referential processing and future planning and disruptions in interoceptive awareness [16]. These studies suggest that NSSI may be related to emotional reactivity, impulsivity control, and reward systems. The current research on NSSI has the following characteristics: approximately 45% of self-harm articles were published in the past three years [11]; the study participants were mostly small samples (less than 50 participants) [15, 17, 18]; a single magnetic resonance imaging technique was used, and only one study involved patients with clinically diagnosed MDD. Thus, the use of larger samples in MDD and multimodal magnetic resonance imaging is necessary to explore the neuroimaging changes of NSSI.

In this study, we explored the potential neurobiological effects of NSSI using resting-state functional magnetic resonance imaging (fMRI) and a three-dimensional T1-weighted structural model combined with a random forest model. We expected to apply multimodal neuroimaging to screen indicators related to NSSI in MDD and build a machine learning model of brain regions with significant discriminative features, which would help to predict NSSI in MDD. In addition, the identification of these location-specific properties will offer us a new target for treatment.

## Method

### Participants and clinical evaluation

A total of 392 participants were recruited at Renmin Hospital of Wuhan University from April to December 2020, including patients from the Early-Warning System and Comprehensive Intervention for Depression (ESCID) study [7]. The samples included 260 MDD patients and 132 healthy controls (HCs). They were all diagnosed by two experienced psychiatrists and met the Diagnostic and Statistical Manual of Mental Disorders, fifth edition, diagnostic criteria for moderate to MDD and were screened with the Mini-International Neuropsychiatric Interview (MINI) [19, 20]. MDD patients were excluded if they were ≤16 years of age or ≥55 years of age, had major neurological or other psychiatric disorders, had magnetic resonance imaging abnormalities, transcranial magnetic stimulation in the last month, or had contraindications. HCs were recruited from the college and the local community using the following criteria: no mood disorders or neurological disorders, no history of substance or alcohol dependence, and no imaging abnormalities or MRI contraindications. This experiment was reviewed and approved by the Ethics Committee of the Renmin Hospital of Wuhan University, and all participants were informed and agreed to participate in this study.

MRI data that included head motion of more than 3.0 mm or an angular rotation greater than 3° in any direction were excluded from the analysis. Additional sequences (T2-weighted and fluid-attenuated inversion recuperation) were acquired and analyzed to rule out concomitant diseases such as ischaemic stroke and susceptibility artefacts from prior haemorrhage or space-occupying lesions. All MDD patients underwent assessment for depression severity by using the 17-item Hamilton Depression Rating Scale (HAMD-17) [21].

After ruling out poor-quality images, 346 participants were retained for MRI analysis (including 235 MDD patients and 111 HCs). Demographic data were self-reported by the participants, including sex, age, depression course, and educational level. Based on the Kiddie-SADS - Lifetime Version (K-SADS-PL) screen interview, which is a common item for NSSI diagnosis for all age groups, we trained clinicians to conduct interviews to determine the history and frequency of NSSI in the participating patients [22]. The specific interview methods and steps were described in our previous study, including: “Did you try to hurt yourself and how?”, “Have you ever cut, burn, or hit (etc.) yourself?”, “Why did you do this? Were you intending to kill yourself or not?”, “How many times have you done this kind of behaviour?” [7].

### MRI acquisition and preprocessing

#### MRI acquisition

MRI scans were obtained using a General Electric Company 3.0 T scanner (GE Discovery MR750 3.0 T) at the Renmin Hospital of Wuhan University Hospital. A localizer sequence was acquired to position subsequent scans, followed by a 4 min-long high-resolution three-dimensional T1-weighted structural scan (repetition time = 8.5 ms; echo time = 3.2 ms; FOV = 25.6*25.6 mm; slice thickness = 1.0 mm). The participants then underwent a 16 min resting-state functional scan with eyes closed while letting their minds wander (spin-echo echo-planar imaging sequence: repetition time = 2000 ms, echo time = 30 ms, flip angle = 90, FOV = 24*24 mm, matrix = 64*64, slice gap = 1 mm, slice thickness = 3.0 mm, slice number = 36).

### fMRI image preprocessing

Data preprocessing was conducted using the Data Processing & Analysis for Brain Imaging (DPARSF, http://restfmri.net/forum/DPARSF) [23] and the Resting-State fMRI Data Analysis Toolkit (REST, http://www.restfmri.net/forum/REST_V1.8). The steps of rs-fMRI preprocessing were as follows: removal of the first 10 volumes, slice timing, realignment, spatial normalization through EPI, linear detrending, and regression of nuisances (grey and white matter signals, cerebrospinal fluid signal, global mean signal and head motion parameters (Friston 24 parameters)). Then, the standardized regional homogeneity analysis (ReHo) map of each participant was calculated. For subsequent statistical analysis, Fisher’s r-to-z transformation was applied to improve the normality of the correlation.

### Functional connectome reconstruction

The graph theoretical topological properties were determined using GRETNA software [24]. The functional connectome was reconstructed for each participant by calculating Pearson correlations of the mean time series between automated anatomical labelling (AAL) 116 atlas nodes, which consists of 90 subregions in the cerebrum and 26 subregions in the cerebellum [25]. Thirty-six sparsity thresholds (0.05–0.4, with an interval of 0.01) were applied to create the binary matrix [26]. The brain topology properties of the functional connectome were calculated. Sigma was computed as the efficiency of segmentation and integration. The clustering coefficient (Cp) indicates the extent of local information segregation [27]. Path length (Lp) measures the capability for information integration. Global efficiency (eg) measures the global efficiency of parallel information transfer in a network. Degree centrality (DC) reflects a node’s communication ability in the functional network. Betweenness centrality (BC) characterizes the node’s effect on information flow between other nodes. The local efficiency (NE) measures the efficiency of communication among the first neighbours of this node when it is removed. After calculating the global and nodal network properties of each sparsity, the AUC (area under the curve) of each patient’s 36 sparsity properties was calculated for further statistical analysis.

### Voxel-based morphometry analysis

SPM 8 and the VBM 8 toolbox (http://dbm.neuro.unijena.de/vbm) were used for T1 image processing and voxel-based morphometry analysis. T1 images were reoriented to have the same point of origin, spatial orientation, and nonlinear deformation field, normalized to a template space and segmented into grey matter, white matter, and cerebrospinal fluid. After the preprocessing step, all images were checked, and normalized images were smoothed with an 8 mm full-width at half-maximum (FWHM) Gaussian kernel. In addition, grey matter volume (GMV) was obtained.

### Statistical Analysis

IBM SPSS Statistics Version 23.0 was used to compare the demographics and clinical characteristics. The following analyses were performed to compare general demographic and mean head motion Jenkinson between MDD patients with NSSI and HCs and MDD patients with and without NSSI. For continuous variables, we used the F test and further used independent sample t tests or Wilcoxon rank-sum tests. SPM12 was used to compare the ReHo and GMV by two-sample t-tests, with a voxelwise *P value* of 0.001 and a familywise error-corrected (FWE) clusterwise *P value* of 0.05. GRETNA was used to compare the AUCs of global and nodal network properties between MDD patients with and without NSSI and HCs (Bonferroni correction). Network-based statistics (NBS) were used to detect functional network differences with edge thresholds of 0.001 and 1000 iteration nonparametric permutation tests [28]. The statistical thresholds were set to *P* < 0.05 for all analyses, with age, sex, depression course, and education level as nuisance covariates. Furthermore, we added HAMD-17 scores to these covariates for further sensitivity analysis. Considering the age-range broad, we made more restrictive age-inclusion and ran an analysis with only participants under 30, including participants younger than 30 years old, with age, sex, depression course, education level, and HAMD-17 scores as nuisance covariates.

### Building a random forest model

The R software randomForest, pROC, caTools package was used to build a random forest model. NSSI history was entered as the predictor variable, and all variables showing statistically significant associations with NSSI were considered; a random forest model with 500 trees was generated. The importance of different data was compared through MeanDecreaseGini [29]. Additionally, 10-fold cross-validation was used, in which each cross-validation test was repeatedly tested using a subset, and the remaining nine subsets were trained. Finally, the best receiver operating characteristic curve (AUC) was obtained [30].

## Results

### Demographic and clinical characteristics

A total of 158 MDD patients without a history of NSSI, 77 MDD patients with a history of NSSI and 111 HCs were retained for analysis. The age distribution of MDD patients with a history of NSSI was lower than that of MDD patients without a history of NSSI and HCs. Specifically, MDD patients with a history of NSSI had a longer depression course than MDD patients without a history of NSSI. There were no sex differences between MDD patients with a history of NSSI and MDD patients without NSSI history and HCs. The two-sample t-test showed significant group effects on education level. The demographic data are shown in Tables 1.

**Table 1:** Demographic data.

Item		Without NSSI	With NSSI	HCs	Without NSSI vs with NSSI	With NSSI vs HC
Age		25.06 ± 6.52	22.09 ± 4.16	26.01 ± 7.41	<0.001	<0.001
Sex	Man	46 (0.54)	15 (0.18)	24 (0.28)	0.114	0.722
	Female	112 (0.43)	62 (0.24)	87 (0.33)		
Education level	Below bachelor	11 (0.42)	8 (0.31)	7 (0.27)	0.575	<0.001
	Bachelor	126 (0.5)	61 (0.24)	64 (0.25)		
	High bachelor	21 (0.3)	8 (0.12)	40 (0.58)		
Oneset age		20.92 ± 6.66	18.26 ± 4.64	NA	0.002	NA
Depression course (month)		29.22 ± 35.62	28.56 ± 24.35	NA	0.884	NA
FD Jenkinson		0.06 ± 0.03	0.06 ± 0.03	0.07 ± 0.04	0.098	0.217

*MDD* major depressive disorder, *NSSI* nonsuicidal self-injury, *HCs* healthy controls.

**Table 2:** Importance ranking of random forest model.

Item	MeanDecreaseAccuracy	MeanDecreaseGini
GMV	0.036694391	27.84020105
Cerebellum-centre network	0.020026355	24.05427538
Reho	0.014789882	22.21512409
Sigma	0.004110613	16.9772032
eg	−0.002983549	11.62811118

*GMV* grey matter volume, *ReHo* regional homogeneity analysis, *eg* global efficiency.

### Regional GMV differences

**Fig. 1:**
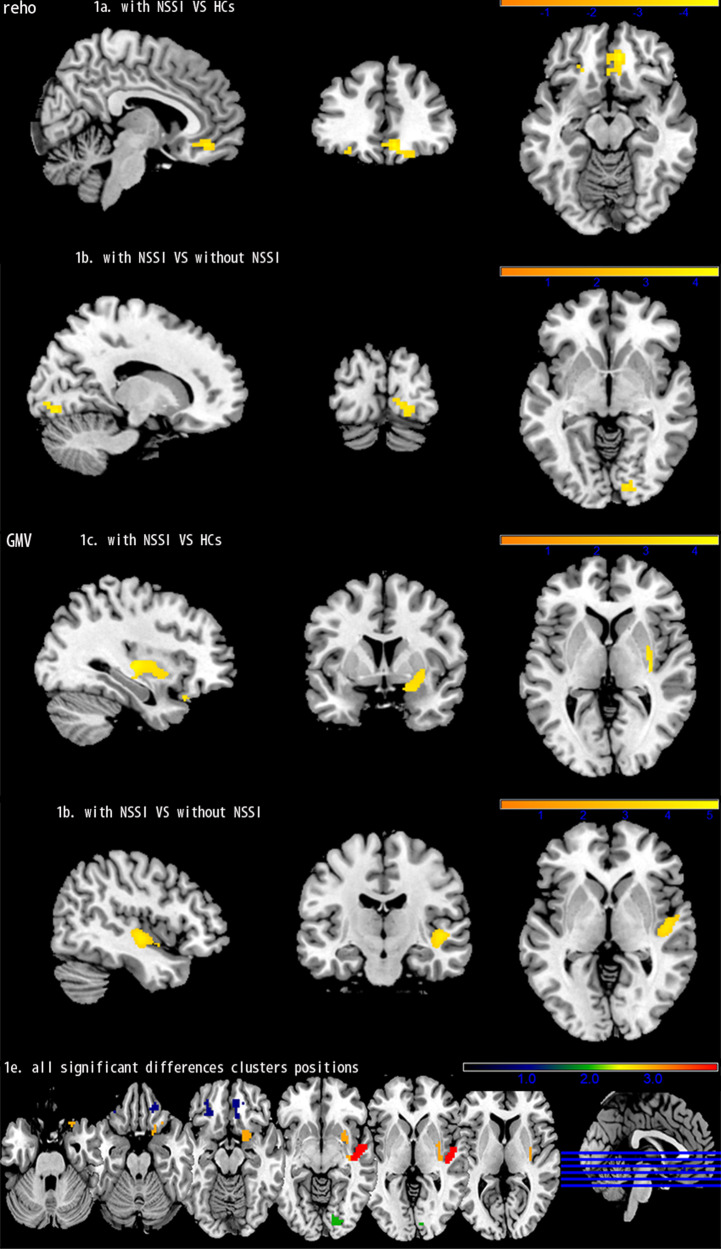
Comparison GMV and ReHo of between MDD with NSSI and HCs, and MDD with and without NSSI. **a**, **b** Show the difference in Reho. **c**, **d** Show the difference in GMV. MDD major depressive disorder, NSSI nonsuicidal self-injury, HCs healthy controls, GMV grey matter volume, ReHo regional homogeneity analysis.

Compared with the HCs, the MDD patients with a history of NSSI showed significant GMV increases in the right putamen, right inferior orbital frontal cortex (OFC), right olfactory cortex, and right amygdala. Compared with the MDD patients without a history of NSSI, the MDD patients with a history of NSSI showed significant GMV increases in the right superior temporal gyrus and right insula. The cluster size, peak T value, and peak MNI coordinates of regions with decreased ReHo are listed in eTable 1 and Fig. 1. After adding HAMD-17 score as a covariate and more restrictive age inclusion, the above brain regions still showed differences, as shown in eTables 8 and 11.

### Regional ReHo differences

Compared with the HCs, the MDD patients with a history of NSSI showed significant ReHo increases in the right median OFC and left median OFC. Compared with the MDD patients without a history of NSSI, the MDD patients with a history of NSSI showed a significant ReHo decrease in the right lingual gyrus. The cluster size, peak T value, and peak MNI coordinates of regions with decreased ReHo are listed in eTable 1 and Fig. 1. After adding HAMD-17 score as a covariate and more restrictive age inclusion, the above brain regions still showed differences, as shown in eTables 8 and 11.

### Global connectome topology

**Fig. 2:**
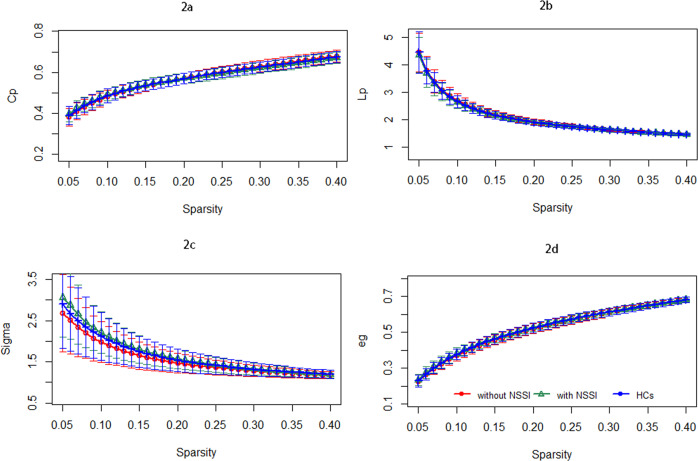
Comparison of global connectome topology differences between different groups. **a**–**d** Comparisons between MDD patients with NSSI and HCs and MDD patients with and without NSSI of Cp, Lp, Sigma and Eg. MDD major depressive disorder, NSSI nonsuicidal self-injury, HCs healthy controls, Cp clustering coefficient, Lp path length, Eg global efficiency.

Across the defined threshold range, both patients and HCs demonstrated small-world topological properties (σ > 1). At the large-scale network level, MDD patients with a history of NSSI showed significantly different sigma and global efficiency compared with MDD patients without a history of NSSI. However, there were no significant differences in Lp and Cp. In the regional topologic organization analysis, compared with HCs, the MDD patients with a history of NSSI exhibited decreased DC and ne in the left pallidum (*P* = 0.0001, *P* = 0.0004, respectively, corrected; eTables 2, 3 and Fig. 2). No significant differences in BC were observed among the groups (*P* > 0.05, Bonferroni correction). After adding HAMD-17 score as a covariate, MDD patients with a history of NSSI showed significantly different sigma compared with MDD patients without a history of NSSI(*P* = 0.011). However, there were no significant differences in Eg (*P* = 0.052), Lp (*P* = 0.094), or Cp (*P* = 0.719). No significant differences in BC, DC, or NE were observed among the groups, as shown in eTable 9. MDD patients with a history of NSSI showed significantly different sigma (*P* = 0.009) and Eg (*P* = 0.022) than MDD patients without a history of NSSI, after controlling for the more restrictive age-inclusion (eTable 12).

**Fig. 3:**
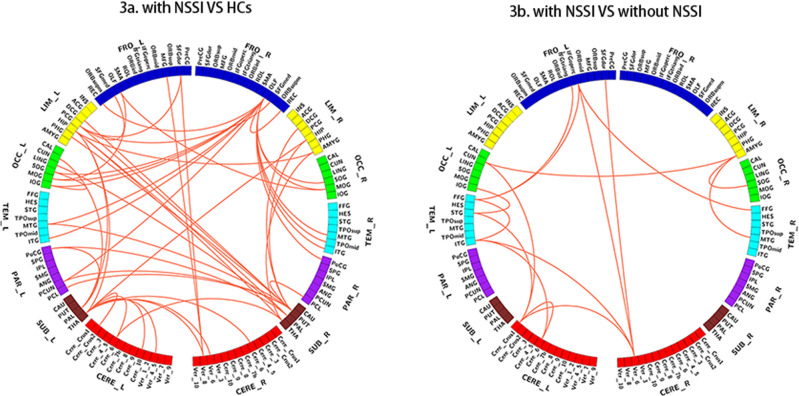
Network-based statistics difference between MDD with NSSI and HCs and MDD with and without NSSI. MDD major depressive disorder, NSSI nonsuicidal self-injury, HCs healthy controls.

Compared with HCs, the MDD patients with a history of NSSI showed significant decreases in the olfactory-centre network (50 edges, 44 nodes), including important nodes in the right olfactory (14 edges), right caudate nucleus (9 edges), left pallidum (9 edges), and left hippocampus (5 edges) (eTable 5 and Fig. 3a). Compared with the MDD patients without a history of NSSI, the MDD patients with NSSI history showed significantly decreased networks in the cerebellum-centre network(16 edges, 15 nodes), including important nodes in the cerebellum (left cerebellum_superior 4 edges, Vermis_6 4 edges), the left inferior frontal gyrus opercular part (3 edges), the right amygdala (3 edges), and the left thalamus (3 edges) (eTable 6 and Fig. 3b). After adding the HAMD-17 score as a covariate, MDD patients with a history of NSSI showed a significantly different network in the cerebellum-centre network (16 edges, 15 nodes), including important nodes in the cerebellum (left cerebellum_superior 4 edges, Vermis_6 4 edges), the left inferior frontal gyrus opercular part (3 edges), the right amygdala (3 edges), and the left thalamus (3 edges), as shown in the eTable 10. After adding more restrictive age-inclusion, the cerebellum-centre network still showed differences, as shown in the eTable 13.

### Random forest model

We used all previously obtained neuroimaging indicators of MDD patients without NSSI and NSSI history as predictors and used NSSI history as a label prediction to construct the model. The importance ranking of the above factors in predicting whether MDD is accompanied by NSSI is shown in Table 2. The GMV of the right STG, cerebellum core network, and ReHo of the right lingual gyrus were more important than other factors. The AUC of the above factors in predicting whether MDD was associated with NSSI was 0.781, with a specificity of 0.625 and a sensitivity of 0.938.

## Discussion

To the best of our knowledge, this is the first study performed as an exploratory analysis using multimodal magnetic resonance imaging to investigate and predict NSSI abnormalities in MDD patients, discovering the mechanisms of NSSI related to MDD. MDD patients with a history of NSSI showed significantly different GMV in the right putamen, right superior OFC ReHo, and DC and NE in the left pallidum than HCs. Compared to MDD patients without a history of NSSI, MDD patients with a history of NSSI had significantly different right STG GMV, right lingual gyrus ReHo, sigma, and global efficiency. The right STG GMV and cerebellum core network were important in predicting whether MDD was accompanied by NSSI. Most differences remained significant after controlling for depression severity and more restrictive age-inclusion. We discuss potential interpretations of these findings below.

Multiple NSSI theoretical models, such as the experiential avoidance model, the emotional cascade model, the cognitive-emotional model and the benefits and barriers model, describe the psychological characteristics of the different stages of NSSI occurrence, including avoiding aversive emotional experiences that perform primarily NSSI, positive and negative reinforcements that explain maintenance impulsiveness, and self-related cognitions that predict NSSI behaviour [31–34]. The brain areas involved in these thought processes may be abnormal. A subsequent NSSI neuroimaging study showed activation and structural changes in the medial PFC, ventrolateral PFC, parahippocampus [35], putamen [35], posterior insula activation [17], anterior insula response [36], and left ACC [37], which reflect that unpleasant somatosensory sensations, emotional reactivity, parasuicidal behaviour, and impulsivity are related to NSSI. Similarly, our research shows that MDD patients with a history of NSSI have abnormalities in the emotional circuit, sensory system, and pain regulation, including the putamen, OFC, pallidum, olfactory cortex, amygdala, insula, and lingual gyrus. Multiple brain regions involving multiple theoretically relevant regions also reflect the diversification and heterogeneity of the occurrence of NSSI, providing greater challenges for the treatment of NSSI.

Emotional circuit and sensory abnormalities are mentioned in NSSI, but the development of pain regulation in NSSI needs further study [36]. In general, pain dissuades most people from engaging in NSSI, and the erosion of this barrier in patients may facilitate NSSI [38]. Feeling pain dysregulated spontaneous activity in regions related to pain regulation and emotional arousal involved in the prefrontal–limbic–midbrain circuit and somatosensory processing [39, 40]. Our research also showed abnormalities in these brain regions, suggesting that patients with MDD with NSSI have abnormal pain regulation. Although pain sensitivity is dynamic, it may tend to normalize after cessation of NSSI [41]. The increase in the frequency of NSSI also increases the patient’s pain tolerance and reduces the efficacy of NSSI in regulating emotions and does not implement NSSI [42]. Although no conclusions can be drawn regarding a causal relationship between pain sensitivity and NSSI, these findings support that pain sensitivity is a proximal feature of NSSI development and termination. The mechanism is still controversial, and the endogenous opioid system and β-endorphin may be involved in NSSI development [43, 44]. Therefore, we also speculate that patients with NSSI may have abnormal pain perception.

In addition, our study first discovered and highlighted the importance of STG GMV and the cerebellar functional connection network in the occurrence of NSSI in MDD. The STG surface areas and functional connectivities were correlated with new MDD episodes and symptomatology [45–48]. Previous research has shown that STG volume could predict violence during the previous 6 months [48]. In our study, the STG GMV change was a predictor of NSSI in MDD patients, and it also supports the important role of the STG in impulsive behaviour. The rostral superior temporal sulcus, the most integrative node of the social cognition network, is involved in fast-paced bottom-up sensorimotor coupling [49]. The STG GMV changes this balance and generates impulsive behaviour. In addition, previous studies reported STG GMV changes in depressive patients with psychotic features [50, 51] and were associated with electroconvulsive therapy improvement [52]. Our previous research also showed the importance of psychiatric symptoms in MDD patients with NSSI. Therefore, we speculate that the mediation between NSSI in MDD and the changes in psychiatric symptoms and the STG still needs to be further confirmed. In addition, our research has shown an increase in STG GMV, while previous studies have shown more of an increase in GMV in MDD. On the one hand, the increase in STG GMV shows that this may be a compensatory mechanism. On the other hand, the change in GMV is one of the changes in grey matter, and the multiangle study of density and other changes provides a comprehensive understanding. It remains to be seen whether the variability of the STG GMV among MDD patients reflects a compensatory mechanism, or rather a causal deficit that leads to NSSI in MDD in this population.

Notably, it is now known that the cerebellum also plays important roles in emotion, cognitive control, fear memory [40] and executive function [53]. Abnormal structure and function in the cerebellum have been reported in MDD patients [54–56]. Structural and functional disruptions in cortico-cerebellar and cerebello-thalamo-cortico loops in patients with MDD are associated with response-inhibition processes [40, 44, 57, 58] and cognitive and motor disturbances frequently observed in MDD [55, 59, 60]. Prefrontal-cerebellar loops regulate the start or end of actions by providing sensory feedback through cerebellar internal models [61] Our research also found cortico-cerebellar loop dysfunction in MDD patients with NSSI. Other studies have also shown that higher scores on the lack of positive affect subscale were related to vermis VIII volumes [62]. These findings highlight the phenotype that may be accompanied by NSSI as a unique clinical heterogeneity. In this process, urbanicity effects and stress processes are connected with the cerebellum [56, 63]. In addition, studies have shown prefrontal-cerebellar loops associated with ketamine treatment response [64, 65]. This study, together with our research, reveals the contribution of cerebrocerebellar circuitries in MDD pathophysiology and highlights the need to further investigate these circuitries and their role in executive control processes in depression and its treatment. In addition, the roles of different areas of the cerebellum in emotion, cognition, and behaviour have gradually been discovered. Our research has enriched this evidence.

### Limitations

This study has some limitations that should be noted. First, this study was a retrospective study with possible recall bias. In addition, we cannot rule out the possibility that the difference in medication intake accounts for some of the differences found in brain morphology, and differences in the types and doses of antidepressants will be further considered in future studies. Fortunately, our study is based on the use of a large number of real-world samples, which should reduce the interference of drug differences but still urges more standardized drug experiments for analysis. Second, our study used a large number of clinically diagnosed MDD patients but did not further decompose current NSSI and the effect of different age on the occurrence of NSSI should be further discussed. At the same time, we focused on attention to the occurrence of NSSI at different ages, and obtained similar changes by narrowing the age range in the data analysis, but the role of age differences in the occurrence of NSSI cannot be ignored and consideration of the interaction between NSSI and the occurrence of MDD in the whole course of the disease [66]. Third, the constructed machine learning model did not undergo longitudinal clinical verification in a larger sample. Fortunately, our study is continuing its follow-up assessments of the remaining individuals; thus, the NSSI group might have a sufficient sample size to address this limitation and test for similar results in the future. Our study found that MDD patients with a history of NSSI have abnormal emotions, pain regulation, and the somatosensory system, but it is still unclear whether there are corresponding changes in clinical symptoms. In the future, the relationship between these symptoms and phenotype should be strengthened to define the corresponding change. At the same time, based on the current exploratory research, more specific hypothesis-driven and multiple templates research should be carried out to explore the mechanism of NSSI.

## Conclusion

MDD patients with a history of NSSI have dysregulated spontaneous brain activity and structure in regions related to emotions, pain regulation, and the somatosensory system. Importantly, right STG GMV and cortico-cerebellar loops may play important roles in NSSI in MDD patients.

## Supplementary information

SUPPLEMENTAL MATERIAL
