# Supplementary material for: Superior temporal gyrus and cerebellar loops predict nonsuicidal self-injury in major depressive disorder patients by multimodal neuroimaging
Source: Transl Psychiatry. 2022 Nov 10;12:474. doi: 10.1038/s41398-022-02235-y (PMC9649804; doi:10.1038/s41398-022-02235-y)
Supplement: Supplementary file 1 — SUPPLEMENTAL MATERIAL [file 41398_2022_2235_MOESM1_ESM.doc]

Part 1

This part set age, sex, depression course and education level as nuisance covariates.

| eTable 1. Regional GMV and ReHo differences | | | | | | | |
| --- | --- | --- | --- | --- | --- | --- | --- |
|  |  | region | cluster size | x | y | z | t |
| ReHo | with NSSI vs HC | right med OFC | 72 | 9 | 42 | -12 | -4.7155 |
|  |  | left med OFC | 28 | -27 | 42 | -15 | -3.8177 |
|  | without NSSI vs with NSSI | right lingual gyrus | 43 | 18 | -81 | -9 | 4.425 |
| GMV | with NSSI vs HC | right putamen | 427 | 31.5 | -16.5 | 4.5 | 4.4414 |
|  |  | right inferior OFC | 159 |  | | | |
|  |  | right olfactory cortex | 111 |  |  |  |  |
|  |  | right amygdala | 43 |  |  |  |  |
|  | without NSSI vs with NSSI | right superior temporal gyrus | 725 | 49.5 | -10.5 | -4.5 | 5.1493 |
|  |  | right insula | 92 |  | | | |
| MDD: major depressive disorder, NSSI: nonsuicidal self-injury, HCs: healthy controls, GMV: grey matter volume; ReHo: regional homogeneity analysis, OFC: orbital frontal cortex | | | | | | | |

| eTable 2. MDD without NSSI vs with NSSI | | | | | | |
| --- | --- | --- | --- | --- | --- | --- |
|  | BC | | DC | | NE | |
| **Regions** | *p* | *t* | *p* | *t* | *p* | *t* |
| left precental gyrus | 7.59E-01 | -3.07E-01 | 5.29E-01 | 6.31E-01 | 9.42E-01 | 7.30E-02 |
| right precental gyrus | 9.28E-01 | 9.03E-02 | 1.30E-01 | 1.52E+00 | 3.92E-01 | 8.58E-01 |
| left superior frontal gyrus, dorsolateral | 2.15E-01 | 1.24E+00 | 9.91E-01 | -1.13E-02 | 7.87E-01 | -2.71E-01 |
| right superior frontal gyrus, dorsolateral | 3.19E-01 | 1.00E+00 | 9.76E-01 | 3.06E-02 | 7.60E-01 | -3.05E-01 |
| left superior frontal gyrus, orbital part | 5.97E-01 | -5.29E-01 | 1.47E-01 | -1.45E+00 | 9.36E-02 | -1.68E+00 |
| right superior frontal gyrus, orbital part | 3.06E-01 | -1.03E+00 | 1.60E-01 | -1.41E+00 | 7.22E-02 | -1.81E+00 |
| left middle frontal gyrus | 4.56E-01 | -7.47E-01 | 6.19E-01 | 4.98E-01 | 8.82E-01 | 1.49E-01 |
| right middle frontal gyrus | 6.72E-01 | -4.24E-01 | 5.78E-01 | 5.57E-01 | 9.33E-01 | 8.36E-02 |
| left middle frontal gyrus, orbital part | 9.57E-02 | 1.67E+00 | 9.63E-01 | 4.70E-02 | 5.50E-01 | -5.99E-01 |
| right middle frontal gyrus, orbital part | 8.99E-01 | -1.27E-01 | 2.71E-01 | -1.10E+00 | 2.15E-01 | -1.24E+00 |
| left inferior frontal gyrus, opercular part | 3.95E-01 | -8.52E-01 | 9.16E-01 | 1.06E-01 | 8.04E-01 | -2.48E-01 |
| right inferior frontal gyrus, opercular part | 3.15E-01 | 1.01E+00 | 6.07E-01 | -5.16E-01 | 3.74E-01 | -8.90E-01 |
| left inferior frontal gyrus, triangular part | 6.97E-01 | 3.89E-01 | 7.01E-01 | 3.84E-01 | 9.14E-01 | 1.08E-01 |
| right inferior frontal gyrus, triangular part | 5.87E-01 | -5.43E-01 | 5.23E-01 | -6.40E-01 | 3.42E-01 | -9.51E-01 |
| left inferior frontal gyrus, orbital part | 7.67E-01 | -2.97E-01 | 6.11E-02 | -1.88E+00 | 5.65E-02 | -1.92E+00 |
| right inferior frontal gyrus, orbital part | 2.23E-01 | 1.22E+00 | 6.30E-01 | -4.82E-01 | 4.69E-01 | -7.26E-01 |
| left rolandic operculum | 7.41E-01 | -3.30E-01 | 8.16E-01 | 2.33E-01 | 7.01E-01 | -3.84E-01 |
| right rolandic operculum | 4.15E-01 | -8.16E-01 | 9.33E-01 | 8.44E-02 | 4.49E-01 | -7.58E-01 |
| left supplementary motor area | 9.41E-01 | -7.47E-02 | 8.77E-01 | -1.54E-01 | 4.27E-01 | -7.95E-01 |
| right supplementary motor area | 9.17E-01 | -1.04E-01 | 9.87E-01 | -1.58E-02 | 3.43E-01 | -9.50E-01 |
| left olfactory cortex | 9.85E-01 | 1.87E-02 | 5.81E-01 | -5.53E-01 | 9.18E-01 | -1.03E-01 |
| right olfactory cortex | 4.18E-01 | -8.11E-01 | 7.70E-01 | -2.93E-01 | 8.61E-01 | -1.75E-01 |
| left superior frontal gyrus, medial | 3.28E-01 | 9.81E-01 | 2.97E-01 | -1.05E+00 | 2.03E-01 | -1.28E+00 |
| right superior frontal gyrus, medial | 3.27E-01 | 9.82E-01 | 6.85E-01 | -4.06E-01 | 4.11E-01 | -8.24E-01 |
| left superior frontal gyrus, medial orbital | 3.28E-01 | 9.80E-01 | 2.04E-01 | -1.27E+00 | 8.74E-02 | -1.72E+00 |
| right superior frontal gyrus, medial orbital | 5.14E-01 | 6.53E-01 | 3.38E-01 | -9.60E-01 | 1.09E-01 | -1.61E+00 |
| left gyrus rectus | 1.01E-01 | -1.65E+00 | 3.04E-03 | -3.00E+00 | 2.16E-03 | -3.10E+00 |
| right gyrus rectus | 1.54E-01 | -1.43E+00 | 3.11E-02 | -2.17E+00 | 1.28E-02 | -2.51E+00 |
| left insula | 7.46E-01 | 3.25E-01 | 6.56E-01 | -4.46E-01 | 4.89E-01 | -6.94E-01 |
| right insula | 9.77E-01 | -2.91E-02 | 7.57E-01 | -3.09E-01 | 3.37E-01 | -9.62E-01 |
| left anterior cingulate and paracingulate gyri | 7.00E-01 | 3.86E-01 | 4.23E-01 | -8.03E-01 | 2.54E-01 | -1.14E+00 |
| right anterior cingulate and paracingulate gyri | 4.38E-01 | 7.77E-01 | 5.54E-01 | -5.93E-01 | 3.77E-01 | -8.85E-01 |
| left median cingulate and paracingulate gyri | 1.38E-01 | 1.49E+00 | 4.56E-02 | 2.01E+00 | 1.03E-01 | 1.64E+00 |
| right median cingulate and paracingulate gyri | 5.43E-01 | 6.10E-01 | 1.69E-01 | 1.38E+00 | 4.52E-01 | 7.54E-01 |
| left posterior cingulate gyrus | 7.98E-02 | -1.76E+00 | 3.59E-01 | -9.18E-01 | 1.00E-01 | -1.65E+00 |
| right posterior cingulate gyrus | 7.36E-01 | 3.38E-01 | 8.37E-01 | 2.06E-01 | 3.97E-01 | -8.48E-01 |
| left hippocampus | 7.15E-01 | 3.66E-01 | 7.16E-01 | -3.64E-01 | 6.29E-01 | -4.83E-01 |
| right hippocampus | 1.08E-01 | -1.61E+00 | 1.42E-01 | -1.47E+00 | 7.51E-02 | -1.79E+00 |
| left parahippocampal gyrus | 4.95E-01 | -6.83E-01 | 3.73E-01 | -8.93E-01 | 2.39E-01 | -1.18E+00 |
| right parahippocampal gyrus | 5.33E-01 | -6.25E-01 | 4.03E-01 | -8.38E-01 | 2.46E-01 | -1.16E+00 |
| left amygdala | 6.70E-01 | -4.27E-01 | 9.99E-01 | -1.07E-03 | 5.68E-01 | -5.72E-01 |
| right amygdala | 4.37E-01 | 7.79E-01 | 7.37E-01 | 3.36E-01 | 5.55E-01 | -5.92E-01 |
| left calcarine fissure and surrounding cortex | 9.75E-01 | -3.14E-02 | 6.39E-01 | 4.70E-01 | 9.55E-01 | -5.68E-02 |
| right calcarine fissure and surrounding cortex | 5.69E-01 | -5.71E-01 | 8.49E-01 | 1.90E-01 | 6.87E-01 | -4.03E-01 |
| left cuneus | 8.34E-01 | 2.10E-01 | 6.95E-01 | 3.92E-01 | 9.48E-01 | 6.50E-02 |
| right cuneus | 7.27E-01 | 3.50E-01 | 8.85E-01 | 1.44E-01 | 8.05E-01 | -2.47E-01 |
| left lingual gyrus | 5.87E-01 | 5.43E-01 | 3.70E-01 | 8.99E-01 | 7.43E-01 | 3.29E-01 |
| right lingual gyrus | 6.05E-01 | 5.18E-01 | 4.62E-01 | 7.36E-01 | 9.04E-01 | 1.21E-01 |
| left superior occipital gyrus | 4.80E-01 | 7.07E-01 | 1.88E-01 | 1.32E+00 | 3.15E-01 | 1.01E+00 |
| right superior occipital gyrus | 8.01E-01 | 2.52E-01 | 8.36E-01 | 2.07E-01 | 9.86E-01 | 1.82E-02 |
| left middle occipital gyrus | 6.21E-01 | 4.95E-01 | 3.94E-01 | 8.53E-01 | 5.97E-01 | 5.29E-01 |
| right middle occipital gyrus | 8.38E-01 | 2.04E-01 | 5.47E-01 | 6.04E-01 | 9.70E-01 | 3.80E-02 |
| left inferior occipital gyrus | 4.76E-01 | -7.15E-01 | 4.03E-01 | 8.37E-01 | 7.83E-01 | 2.76E-01 |
| right inferior occipital gyrus | 2.16E-01 | -1.24E+00 | 9.89E-01 | -1.39E-02 | 6.29E-01 | -4.84E-01 |
| left fusiform gyrus | 1.88E-01 | 1.32E+00 | 1.95E-01 | 1.30E+00 | 6.19E-01 | 4.98E-01 |
| right fusiform gyrus | 6.68E-02 | -1.84E+00 | 3.62E-01 | -9.14E-01 | 1.17E-01 | -1.57E+00 |
| left postcentral gyrus | 3.10E-01 | -1.02E+00 | 3.99E-01 | 8.45E-01 | 7.27E-01 | 3.49E-01 |
| right postcentral gyrus | 6.49E-01 | -4.56E-01 | 7.28E-01 | 3.48E-01 | 7.60E-01 | -3.06E-01 |
| left superior parietal gyrus | 6.60E-01 | -4.41E-01 | 3.86E-01 | 8.68E-01 | 7.40E-01 | 3.33E-01 |
| right superior parietal gyrus | 1.10E-01 | -1.60E+00 | 3.41E-01 | -9.55E-01 | 8.30E-02 | -1.74E+00 |
| left inferior parietal, but supramarginal and angular gyri | 1.63E-01 | 1.40E+00 | 1.43E-02 | 2.47E+00 | 3.91E-02 | 2.08E+00 |
| right inferior parietal, but supramarginal and angular gyri | 8.81E-01 | 1.50E-01 | 9.26E-01 | -9.35E-02 | 8.46E-01 | -1.95E-01 |
| left supramarginal gyrus | 2.63E-01 | 1.12E+00 | 2.05E-01 | 1.27E+00 | 3.14E-01 | 1.01E+00 |
| right supramarginal gyrus | 4.05E-01 | -8.34E-01 | 7.73E-01 | -2.89E-01 | 3.79E-01 | -8.81E-01 |
| left angular gyrus | 4.75E-01 | 7.15E-01 | 2.48E-02 | 2.26E+00 | 1.14E-01 | 1.58E+00 |
| right angular gyrus | 8.64E-01 | 1.72E-01 | 9.29E-01 | 8.95E-02 | 9.75E-01 | -3.18E-02 |
| left precuneus | 7.59E-02 | -1.78E+00 | 6.99E-01 | -3.88E-01 | 4.81E-01 | -7.05E-01 |
| right precuneus | 1.48E-01 | -1.45E+00 | 4.38E-01 | -7.77E-01 | 2.04E-01 | -1.27E+00 |
| left paracentral lobule | 4.23E-01 | -8.02E-01 | 6.18E-01 | 5.00E-01 | 9.78E-01 | 2.81E-02 |
| right paracentral lobule | 2.08E-01 | -1.26E+00 | 8.41E-01 | -2.01E-01 | 3.46E-01 | -9.44E-01 |
| left caudate nucleus | 4.16E-02 | -2.05E+00 | 5.79E-01 | -5.56E-01 | 6.24E-01 | -4.91E-01 |
| right caudate nucleus | 9.41E-01 | 7.38E-02 | 7.67E-01 | 2.96E-01 | 9.28E-01 | -9.01E-02 |
| left lenticular nucleus, putamen | 6.47E-01 | 4.59E-01 | 5.94E-01 | 5.33E-01 | 8.94E-01 | 1.33E-01 |
| right lenticular nucleus, putamen | 1.74E-02 | -2.39E+00 | 3.03E-01 | -1.03E+00 | 2.12E-01 | -1.25E+00 |
| left lenticular nucleus, pallidum | 1.06E-01 | -1.62E+00 | 6.24E-01 | -4.91E-01 | 7.84E-01 | -2.75E-01 |
| right lenticular nucleus, pallidum | 3.46E-03 | -2.95E+00 | 2.95E-01 | -1.05E+00 | 3.05E-01 | -1.03E+00 |
| left thalamus | 9.74E-02 | 1.66E+00 | 1.89E-01 | 1.32E+00 | 4.59E-01 | 7.42E-01 |
| right thalamus | 5.23E-02 | 1.95E+00 | 3.90E-01 | 8.62E-01 | 6.81E-01 | 4.11E-01 |
| left heschl gyrus | 7.38E-02 | -1.80E+00 | 2.08E-01 | -1.26E+00 | 7.51E-02 | -1.79E+00 |
| right heschl gyrus | 1.01E-03 | -3.33E+00 | 2.93E-02 | -2.19E+00 | 2.48E-02 | -2.26E+00 |
| left superior temporal gyrus | 9.89E-01 | -1.38E-02 | 1.24E-01 | 1.54E+00 | 3.28E-01 | 9.80E-01 |
| right superior temporal gyrus | 6.17E-01 | 5.01E-01 | 5.53E-01 | 5.94E-01 | 9.54E-01 | 5.73E-02 |
| left temporal pole: superior temporal gyrus | 9.00E-01 | 1.26E-01 | 8.48E-01 | 1.92E-01 | 6.68E-01 | -4.29E-01 |
| right temporal pole: superior temporal gyrus | 9.83E-01 | 2.19E-02 | 7.72E-01 | 2.90E-01 | 7.78E-01 | -2.82E-01 |
| left middle temporal gyrus | 3.81E-01 | 8.78E-01 | 2.99E-02 | 2.18E+00 | 5.74E-02 | 1.91E+00 |
| right middle temporal gyrus | 3.03E-01 | 1.03E+00 | 5.32E-01 | 6.26E-01 | 6.55E-01 | 4.48E-01 |
| left temporal pole: middle temporal gyrus | 9.90E-01 | -1.22E-02 | 5.78E-01 | -5.56E-01 | 2.60E-01 | -1.13E+00 |
| right temporal pole: middle temporal gyrus | 3.60E-01 | -9.17E-01 | 1.37E-01 | -1.49E+00 | 7.48E-02 | -1.79E+00 |
| left inferior temporal gyrus | 6.43E-01 | 4.65E-01 | 2.22E-01 | 1.22E+00 | 3.87E-01 | 8.66E-01 |
| right inferior temporal gyrus | 2.83E-01 | -1.08E+00 | 9.39E-01 | -7.63E-02 | 6.60E-01 | -4.40E-01 |
| Cerebelum_Crus1_L | 9.57E-01 | 5.39E-02 | 1.56E-01 | 1.42E+00 | 4.11E-01 | 8.24E-01 |
| Cerebelum_Crus1_R | 7.74E-01 | 2.87E-01 | 5.15E-01 | 6.53E-01 | 8.85E-01 | 1.45E-01 |
| Cerebelum_Crus2_L | 4.48E-01 | -7.60E-01 | 8.57E-01 | 1.81E-01 | 9.88E-01 | 1.52E-02 |
| Cerebelum_Crus2_R | 5.44E-02 | 1.93E+00 | 2.82E-01 | 1.08E+00 | 4.95E-01 | 6.83E-01 |
| Cerebelum_3_L | 7.36E-01 | 3.37E-01 | 7.59E-01 | -3.07E-01 | 4.29E-01 | -7.92E-01 |
| Cerebelum_3_R | 4.07E-01 | -8.30E-01 | 2.07E-01 | -1.27E+00 | 2.30E-01 | -1.20E+00 |
| Cerebelum_4_5_L | 3.77E-01 | 8.85E-01 | 6.73E-01 | 4.23E-01 | 9.35E-01 | 8.23E-02 |
| Cerebelum_4_5_R | 3.14E-01 | 1.01E+00 | 8.96E-01 | 1.30E-01 | 8.25E-01 | -2.22E-01 |
| Cerebelum_6_L | 9.69E-01 | -3.84E-02 | 1.17E-01 | 1.57E+00 | 3.47E-01 | 9.43E-01 |
| Cerebelum_6_R | 1.58E-01 | -1.42E+00 | 7.26E-01 | 3.51E-01 | 9.38E-01 | 7.79E-02 |
| Cerebelum_7b_L | 3.77E-01 | -8.85E-01 | 8.20E-01 | -2.28E-01 | 8.74E-01 | -1.58E-01 |
| Cerebelum_7b_R | 8.89E-01 | 1.39E-01 | 7.58E-01 | -3.08E-01 | 4.05E-01 | -8.35E-01 |
| Cerebelum_8_L | 2.52E-01 | -1.15E+00 | 7.50E-01 | 3.19E-01 | 8.41E-01 | 2.01E-01 |
| Cerebelum_8_R | 4.84E-01 | -7.01E-01 | 8.78E-01 | -1.54E-01 | 4.74E-01 | -7.18E-01 |
| Cerebelum_9_L | 2.55E-01 | -1.14E+00 | 9.72E-01 | 3.48E-02 | 7.39E-01 | -3.34E-01 |
| Cerebelum_9_R | 2.07E-01 | -1.27E+00 | 7.75E-01 | -2.86E-01 | 5.91E-01 | -5.38E-01 |
| Cerebelum_10_L | 9.52E-01 | 6.09E-02 | 3.35E-01 | -9.67E-01 | 4.41E-02 | -2.02E+00 |
| Cerebelum_10_R | 9.82E-02 | -1.66E+00 | 3.46E-01 | -9.43E-01 | 1.87E-01 | -1.32E+00 |
| Vermis_1_2 | 2.61E-01 | -1.13E+00 | 1.20E-01 | -1.56E+00 | 3.28E-02 | -2.15E+00 |
| Vermis_3 | 9.71E-01 | 3.61E-02 | 4.00E-01 | -8.44E-01 | 3.16E-01 | -1.00E+00 |
| Vermis_4_5 | 2.37E-01 | -1.19E+00 | 5.86E-01 | -5.46E-01 | 1.57E-01 | -1.42E+00 |
| Vermis_6 | 4.92E-01 | 6.89E-01 | 3.76E-02 | 2.09E+00 | 2.44E-01 | 1.17E+00 |
| Vermis_7 | 4.48E-01 | 7.61E-01 | 6.91E-01 | 3.97E-01 | 8.66E-01 | 1.68E-01 |
| Vermis_8 | 6.22E-01 | -4.93E-01 | 6.05E-01 | -5.17E-01 | 5.34E-01 | -6.23E-01 |
| Vermis_9 | 4.22E-01 | 8.04E-01 | 9.00E-01 | 1.26E-01 | 9.59E-01 | -5.08E-02 |
| Vermis_10 | 6.63E-01 | 4.37E-01 | 8.38E-01 | -2.04E-01 | 1.27E-01 | -1.53E+00 |
| DC: Degree centrality, BC: Betweenness centrality, NE: local efficiency | | | | | | |

| eTable 3. MDD with NSSI vs HCs | | | | | | |
| --- | --- | --- | --- | --- | --- | --- |
|  | BC | | DC | | NE | |
| **Regions** | *p* | *t* | *p* | *t* | *p* | *t* |
| left precental gyrus | 1.37E-01 | 1.49E+00 | 1.17E-01 | 1.57E+00 | 8.97E-02 | 1.70E+00 |
| right precental gyrus | 6.92E-01 | 3.96E-01 | 1.04E-01 | 1.63E+00 | 8.82E-02 | 1.71E+00 |
| left superior frontal gyrus, dorsolateral | 9.14E-01 | 1.09E-01 | 8.26E-01 | -2.21E-01 | 9.87E-01 | -1.68E-02 |
| right superior frontal gyrus, dorsolateral | 9.27E-01 | -9.23E-02 | 5.45E-01 | -6.06E-01 | 5.50E-01 | -5.98E-01 |
| left superior frontal gyrus, orbital part | 2.09E-01 | -1.26E+00 | 1.19E-01 | -1.56E+00 | 1.63E-01 | -1.40E+00 |
| right superior frontal gyrus, orbital part | 2.52E-01 | -1.15E+00 | 1.44E-01 | -1.46E+00 | 1.61E-01 | -1.41E+00 |
| left middle frontal gyrus | 8.53E-01 | 1.86E-01 | 5.39E-01 | 6.15E-01 | 6.78E-01 | 4.16E-01 |
| right middle frontal gyrus | 8.39E-01 | -2.04E-01 | 4.03E-01 | 8.38E-01 | 5.92E-01 | 5.36E-01 |
| left middle frontal gyrus, orbital part | 6.50E-01 | 4.55E-01 | 6.38E-01 | -4.70E-01 | 7.37E-01 | -3.36E-01 |
| right middle frontal gyrus, orbital part | 2.05E-01 | 1.27E+00 | 5.73E-01 | 5.64E-01 | 7.33E-01 | 3.42E-01 |
| left inferior frontal gyrus, opercular part | 4.80E-01 | 7.07E-01 | 6.38E-02 | 1.86E+00 | 9.35E-02 | 1.68E+00 |
| right inferior frontal gyrus, opercular part | 1.84E-01 | 1.33E+00 | 4.83E-01 | 7.02E-01 | 7.50E-01 | 3.19E-01 |
| left inferior frontal gyrus, triangular part | 2.56E-01 | 1.14E+00 | 2.99E-01 | 1.04E+00 | 4.42E-01 | 7.70E-01 |
| right inferior frontal gyrus, triangular part | 9.08E-01 | -1.16E-01 | 9.20E-01 | -1.01E-01 | 7.31E-01 | -3.44E-01 |
| left inferior frontal gyrus, orbital part | 9.85E-01 | -1.89E-02 | 8.60E-01 | -1.76E-01 | 6.40E-01 | -4.68E-01 |
| right inferior frontal gyrus, orbital part | 1.61E-01 | 1.41E+00 | 1.05E-01 | 1.63E+00 | 2.09E-01 | 1.26E+00 |
| left rolandic operculum | 8.30E-01 | 2.15E-01 | 9.04E-01 | 1.21E-01 | 4.66E-01 | 7.30E-01 |
| right rolandic operculum | 2.80E-01 | 1.08E+00 | 9.68E-01 | -4.02E-02 | 6.53E-01 | -4.50E-01 |
| left supplementary motor area | 1.27E-01 | -1.53E+00 | 6.93E-01 | -3.95E-01 | 5.94E-01 | -5.33E-01 |
| right supplementary motor area | 7.05E-01 | 3.79E-01 | 5.66E-01 | 5.75E-01 | 5.83E-01 | 5.50E-01 |
| left olfactory cortex | 6.90E-03 | -2.72E+00 | 2.21E-03 | -3.09E+00 | 1.69E-02 | -2.40E+00 |
| right olfactory cortex | 4.17E-01 | -8.13E-01 | 3.09E-01 | -1.02E+00 | 1.36E-01 | -1.50E+00 |
| left superior frontal gyrus, medial | 1.26E-01 | 1.53E+00 | 3.11E-01 | -1.02E+00 | 3.69E-01 | -9.01E-01 |
| right superior frontal gyrus, medial | 2.64E-02 | 2.23E+00 | 8.28E-01 | -2.17E-01 | 7.14E-01 | -3.67E-01 |
| left superior frontal gyrus, medial orbital | 3.05E-01 | -1.03E+00 | 2.79E-02 | -2.21E+00 | 2.02E-02 | -2.34E+00 |
| right superior frontal gyrus, medial orbital | 8.13E-01 | -2.37E-01 | 1.14E-01 | -1.59E+00 | 5.28E-02 | -1.94E+00 |
| left gyrus rectus | 4.20E-01 | -8.08E-01 | 7.73E-02 | -1.77E+00 | 5.68E-02 | -1.91E+00 |
| right gyrus rectus | 3.41E-01 | -9.55E-01 | 2.24E-01 | -1.22E+00 | 1.16E-01 | -1.58E+00 |
| left insula | 6.65E-01 | 4.34E-01 | 1.73E-01 | 1.37E+00 | 9.28E-02 | 1.69E+00 |
| right insula | 2.06E-01 | 1.27E+00 | 1.07E-01 | 1.62E+00 | 2.22E-01 | 1.22E+00 |
| left anterior cingulate and paracingulate gyri | 6.53E-01 | 4.50E-01 | 5.77E-01 | -5.59E-01 | 4.33E-01 | -7.85E-01 |
| right anterior cingulate and paracingulate gyri | 7.37E-01 | 3.36E-01 | 1.95E-01 | -1.30E+00 | 2.15E-01 | -1.24E+00 |
| left median cingulate and paracingulate gyri | 6.47E-01 | 4.58E-01 | 1.97E-01 | 1.29E+00 | 2.79E-01 | 1.09E+00 |
| right median cingulate and paracingulate gyri | 5.36E-01 | 6.20E-01 | 1.92E-01 | 1.31E+00 | 4.11E-01 | 8.23E-01 |
| left posterior cingulate gyrus | 2.52E-01 | -1.15E+00 | 1.05E-02 | -2.58E+00 | 3.82E-03 | -2.92E+00 |
| right posterior cingulate gyrus | 5.18E-01 | -6.48E-01 | 2.56E-01 | -1.14E+00 | 1.09E-01 | -1.61E+00 |
| left hippocampus | 3.64E-01 | -9.09E-01 | 1.33E-02 | -2.49E+00 | 4.27E-02 | -2.04E+00 |
| right hippocampus | 9.01E-01 | -1.25E-01 | 5.14E-01 | -6.53E-01 | 4.03E-01 | -8.37E-01 |
| left parahippocampal gyrus | 1.31E-01 | -1.51E+00 | 2.72E-02 | -2.22E+00 | 6.21E-02 | -1.87E+00 |
| right parahippocampal gyrus | 9.22E-01 | 9.83E-02 | 3.89E-01 | -8.63E-01 | 2.96E-01 | -1.05E+00 |
| left amygdala | 2.66E-01 | -1.11E+00 | 1.75E-01 | -1.36E+00 | 2.06E-02 | -2.33E+00 |
| right amygdala | 2.43E-01 | 1.17E+00 | 4.61E-01 | -7.37E-01 | 7.92E-02 | -1.76E+00 |
| left calcarine fissure and surrounding cortex | 5.62E-01 | -5.80E-01 | 9.59E-01 | -5.12E-02 | 7.96E-01 | -2.59E-01 |
| right calcarine fissure and surrounding cortex | 8.47E-01 | -1.93E-01 | 8.06E-01 | 2.45E-01 | 9.13E-01 | 1.10E-01 |
| left cuneus | 3.50E-01 | -9.36E-01 | 4.46E-01 | 7.64E-01 | 4.90E-01 | 6.91E-01 |
| right cuneus | 8.02E-01 | 2.51E-01 | 7.61E-01 | 3.05E-01 | 7.36E-01 | 3.38E-01 |
| left lingual gyrus | 4.75E-01 | -7.15E-01 | 7.86E-01 | 2.71E-01 | 9.37E-01 | 7.97E-02 |
| right lingual gyrus | 3.00E-01 | 1.04E+00 | 1.69E-01 | 1.38E+00 | 2.23E-01 | 1.22E+00 |
| left superior occipital gyrus | 9.53E-01 | 5.90E-02 | 1.56E-01 | 1.42E+00 | 2.40E-01 | 1.18E+00 |
| right superior occipital gyrus | 9.12E-01 | 1.11E-01 | 8.27E-01 | 2.18E-01 | 9.07E-01 | 1.17E-01 |
| left middle occipital gyrus | 7.40E-01 | 3.33E-01 | 7.71E-01 | 2.91E-01 | 9.33E-01 | 8.40E-02 |
| right middle occipital gyrus | 6.10E-01 | -5.11E-01 | 3.77E-01 | -8.85E-01 | 2.22E-01 | -1.22E+00 |
| left inferior occipital gyrus | 4.92E-01 | -6.89E-01 | 9.85E-01 | 1.93E-02 | 7.54E-01 | -3.14E-01 |
| right inferior occipital gyrus | 8.87E-01 | 1.42E-01 | 2.32E-01 | 1.20E+00 | 2.54E-01 | 1.14E+00 |
| left fusiform gyrus | 2.41E-01 | -1.18E+00 | 1.78E-01 | -1.35E+00 | 1.17E-01 | -1.57E+00 |
| right fusiform gyrus | 9.45E-01 | -6.94E-02 | 3.93E-01 | -8.55E-01 | 2.39E-01 | -1.18E+00 |
| left postcentral gyrus | 4.92E-01 | -6.88E-01 | 7.97E-01 | 2.57E-01 | 8.52E-01 | 1.87E-01 |
| right postcentral gyrus | 3.36E-01 | -9.64E-01 | 9.33E-01 | 8.45E-02 | 9.36E-01 | -8.09E-02 |
| left superior parietal gyrus | 8.18E-04 | -3.39E+00 | 8.95E-01 | 1.33E-01 | 7.11E-01 | 3.71E-01 |
| right superior parietal gyrus | 2.28E-02 | -2.29E+00 | 2.07E-01 | -1.27E+00 | 1.51E-01 | -1.44E+00 |
| left inferior parietal, but supramarginal and angular gyri | 5.77E-01 | 5.59E-01 | 5.18E-03 | 2.82E+00 | 3.10E-03 | 2.99E+00 |
| right inferior parietal, but supramarginal and angular gyri | 7.01E-01 | -3.85E-01 | 1.04E-01 | 1.63E+00 | 7.71E-02 | 1.77E+00 |
| left supramarginal gyrus | 8.59E-02 | 1.72E+00 | 1.17E-01 | 1.57E+00 | 9.77E-02 | 1.66E+00 |
| right supramarginal gyrus | 7.90E-01 | 2.67E-01 | 2.73E-01 | 1.10E+00 | 3.04E-01 | 1.03E+00 |
| left angular gyrus | 3.93E-02 | 2.07E+00 | 8.96E-02 | 1.70E+00 | 1.54E-01 | 1.43E+00 |
| right angular gyrus | 7.68E-01 | 2.95E-01 | 4.47E-01 | 7.62E-01 | 3.63E-01 | 9.12E-01 |
| left precuneus | 7.07E-01 | -3.76E-01 | 9.59E-01 | 5.15E-02 | 9.22E-01 | 9.80E-02 |
| right precuneus | 8.91E-01 | 1.37E-01 | 7.95E-01 | -2.60E-01 | 7.79E-01 | -2.81E-01 |
| left paracentral lobule | 6.36E-01 | -4.74E-01 | 9.50E-01 | 6.25E-02 | 9.05E-01 | -1.19E-01 |
| right paracentral lobule | 3.75E-01 | -8.88E-01 | 6.73E-01 | -4.23E-01 | 6.26E-01 | -4.88E-01 |
| left caudate nucleus | 2.05E-01 | -1.27E+00 | 1.25E-02 | -2.52E+00 | 1.83E-01 | -1.34E+00 |
| right caudate nucleus | 6.71E-01 | -4.25E-01 | 3.95E-01 | -8.51E-01 | 4.63E-01 | -7.34E-01 |
| left lenticular nucleus, putamen | 2.64E-01 | 1.12E+00 | 1.64E-01 | -1.39E+00 | 2.36E-01 | -1.19E+00 |
| right lenticular nucleus, putamen | 2.74E-01 | 1.10E+00 | 1.74E-01 | -1.36E+00 | 1.41E-01 | -1.48E+00 |
| left lenticular nucleus, pallidum | 2.87E-03 | -3.01E+00 | 1.40E-04 | -3.86E+00 | 4.23E-04 | -3.57E+00 |
| right lenticular nucleus, pallidum | 3.28E-02 | -2.15E+00 | 7.28E-04 | -3.42E+00 | 3.78E-03 | -2.92E+00 |
| left thalamus | 1.11E-01 | 1.60E+00 | 2.05E-01 | 1.27E+00 | 1.67E-01 | 1.39E+00 |
| right thalamus | 1.58E-01 | 1.42E+00 | 2.34E-01 | 1.19E+00 | 1.78E-01 | 1.35E+00 |
| left heschl gyrus | 1.51E-01 | 1.44E+00 | 3.95E-01 | 8.51E-01 | 5.23E-01 | 6.40E-01 |
| right heschl gyrus | 7.36E-01 | -3.38E-01 | 6.49E-01 | -4.55E-01 | 6.00E-01 | -5.26E-01 |
| left superior temporal gyrus | 8.81E-02 | 1.71E+00 | 5.53E-03 | 2.80E+00 | 6.05E-03 | 2.77E+00 |
| right superior temporal gyrus | 6.29E-02 | 1.87E+00 | 9.06E-02 | 1.70E+00 | 7.15E-02 | 1.81E+00 |
| left temporal pole: superior temporal gyrus | 7.75E-01 | -2.86E-01 | 3.01E-01 | 1.04E+00 | 3.42E-01 | 9.51E-01 |
| right temporal pole: superior temporal gyrus | 6.28E-01 | -4.85E-01 | 2.67E-01 | 1.11E+00 | 1.73E-01 | 1.37E+00 |
| left middle temporal gyrus | 4.67E-01 | 7.29E-01 | 6.75E-01 | 4.20E-01 | 5.73E-01 | 5.64E-01 |
| right middle temporal gyrus | 2.53E-02 | 2.25E+00 | 2.57E-01 | 1.14E+00 | 2.19E-01 | 1.23E+00 |
| left temporal pole: middle temporal gyrus | 2.27E-01 | -1.21E+00 | 8.66E-01 | -1.69E-01 | 9.44E-01 | -7.04E-02 |
| right temporal pole: middle temporal gyrus | 4.08E-01 | -8.28E-01 | 2.91E-01 | -1.06E+00 | 1.89E-01 | -1.32E+00 |
| left inferior temporal gyrus | 9.88E-01 | 1.56E-02 | 5.66E-01 | 5.75E-01 | 6.53E-01 | 4.50E-01 |
| right inferior temporal gyrus | 6.52E-01 | 4.52E-01 | 7.23E-01 | 3.55E-01 | 8.56E-01 | 1.82E-01 |
| Cerebelum_Crus1_L | 7.34E-02 | -1.80E+00 | 7.14E-01 | 3.67E-01 | 1.00E+00 | 3.56E-04 |
| Cerebelum_Crus1_R | 2.15E-01 | -1.24E+00 | 8.60E-01 | 1.76E-01 | 8.99E-01 | -1.27E-01 |
| Cerebelum_Crus2_L | 7.72E-01 | -2.90E-01 | 4.78E-01 | 7.11E-01 | 5.86E-01 | 5.45E-01 |
| Cerebelum_Crus2_R | 8.51E-01 | 1.88E-01 | 4.28E-01 | 7.94E-01 | 4.96E-01 | 6.82E-01 |
| Cerebelum_3_L | 9.43E-01 | -7.10E-02 | 7.65E-01 | 2.99E-01 | 4.62E-01 | 7.37E-01 |
| Cerebelum_3_R | 2.52E-01 | -1.15E+00 | 8.72E-01 | 1.61E-01 | 6.39E-01 | 4.70E-01 |
| Cerebelum_4_5_L | 5.75E-01 | 5.61E-01 | 6.35E-01 | 4.75E-01 | 6.29E-01 | 4.83E-01 |
| Cerebelum_4_5_R | 3.94E-01 | 8.53E-01 | 3.61E-01 | 9.16E-01 | 3.81E-01 | 8.78E-01 |
| Cerebelum_6_L | 4.10E-01 | -8.25E-01 | 3.91E-01 | 8.60E-01 | 6.02E-01 | 5.21E-01 |
| Cerebelum_6_R | 3.50E-01 | -9.37E-01 | 8.88E-01 | 1.40E-01 | 8.98E-01 | -1.29E-01 |
| Cerebelum_7b_L | 2.92E-01 | 1.06E+00 | 4.80E-01 | 7.07E-01 | 6.93E-01 | 3.96E-01 |
| Cerebelum_7b_R | 4.09E-01 | 8.26E-01 | 2.02E-01 | 1.28E+00 | 3.38E-01 | 9.59E-01 |
| Cerebelum_8_L | 3.18E-01 | -1.00E+00 | 5.50E-01 | -5.98E-01 | 3.60E-01 | -9.17E-01 |
| Cerebelum_8_R | 1.48E-01 | -1.45E+00 | 7.28E-01 | -3.49E-01 | 5.00E-01 | -6.76E-01 |
| Cerebelum_9_L | 2.01E-01 | -1.28E+00 | 7.16E-01 | -3.64E-01 | 8.77E-01 | -1.55E-01 |
| Cerebelum_9_R | 4.42E-02 | -2.02E+00 | 4.74E-01 | -7.18E-01 | 9.90E-01 | -1.27E-02 |
| Cerebelum_10_L | 3.29E-01 | -9.78E-01 | 2.58E-01 | -1.13E+00 | 8.00E-02 | -1.76E+00 |
| Cerebelum_10_R | 3.01E-03 | -2.99E+00 | 5.38E-01 | -6.16E-01 | 5.56E-01 | -5.89E-01 |
| Vermis_1_2 | 9.72E-01 | -3.50E-02 | 2.47E-01 | -1.16E+00 | 2.31E-01 | -1.20E+00 |
| Vermis_3 | 6.43E-02 | 1.86E+00 | 5.43E-01 | 6.10E-01 | 5.27E-01 | 6.33E-01 |
| Vermis_4_5 | 7.94E-01 | 2.62E-01 | 5.75E-01 | -5.61E-01 | 2.09E-01 | -1.26E+00 |
| Vermis_6 | 6.19E-01 | 4.98E-01 | 1.99E-01 | 1.29E+00 | 5.11E-01 | 6.58E-01 |
| Vermis_7 | 8.31E-01 | 2.14E-01 | 8.07E-01 | 2.44E-01 | 9.59E-01 | 5.18E-02 |
| Vermis_8 | 6.10E-01 | 5.11E-01 | 8.58E-01 | 1.79E-01 | 8.63E-01 | -1.73E-01 |
| Vermis_9 | 7.23E-03 | 2.71E+00 | 1.64E-01 | 1.39E+00 | 9.50E-02 | 1.68E+00 |
| Vermis_10 | 2.59E-01 | 1.13E+00 | 8.19E-01 | -2.29E-01 | 5.74E-01 | -5.62E-01 |
| DC: Degree centrality, BC: Betweenness centrality, NE: local efficiency | | | | | | |

| eTable 5. MDD with NSSI vs HCs | |
| --- | --- |
| Regions | Number |
| right olfactory cortex | 14 |
| right caudate nucleus | 9 |
| left lenticular nucleus, pallidum | 9 |
| left hippocampus | 5 |
| left posterior cingulate gyrus | 4 |
| left lenticular nucleus, putamen | 3 |
| right lenticular nucleus, putamen | 3 |
| right lenticular nucleus, pallidum | 3 |
| Vermis_8 | 3 |
| left superior frontal gyrus, dorsolateral | 2 |
| left olfactory cortex | 2 |
| right hippocampus | 2 |
| right amygdala | 2 |
| left superior occipital gyrus | 2 |
| left middle occipital gyrus | 2 |
| right middle occipital gyrus | 2 |
| left inferior occipital gyrus | 2 |
| left fusiform gyrus | 2 |
| left angular gyrus | 2 |
| left middle temporal gyrus | 2 |
| Cerebelum_6_L | 2 |
| left middle frontal gyrus | 1 |
| left superior frontal gyrus, medial | 1 |
| right superior frontal gyrus, medial | 1 |
| left gyrus rectus | 1 |
| right gyrus rectus | 1 |
| left median cingulate and paracingulate gyri | 1 |
| right median cingulate and paracingulate gyri | 1 |
| left parahippocampal gyrus | 1 |
| left cuneus | 1 |
| left lingual gyrus | 1 |
| right superior occipital gyrus | 1 |
| right fusiform gyrus | 1 |
| left precuneus | 1 |
| right precuneus | 1 |
| right middle temporal gyrus | 1 |
| right temporal pole: middle temporal gyrus | 1 |
| left inferior temporal gyrus | 1 |
| right inferior temporal gyrus | 1 |
| Cerebelum_Crus2_L | 1 |
| Cerebelum_6_R | 1 |
| Cerebelum_10_L | 1 |
| Vermis_4_5 | 1 |
| Vermis_6 | 1 |

| eTable 6. MDD without NSSI vs with NSSI | |
| --- | --- |
| Regions | Number |
| Cerebelum_Crus1_L | 4 |
| Vermis_6 | 4 |
| left inferior frontal gyrus, opercular part | 3 |
| right amygdala | 3 |
| left thalamus | 3 |
| left inferior temporal gyrus | 3 |
| left cuneus | 2 |
| left superior temporal gyrus | 2 |
| Cerebelum_6_L | 2 |
| left superior frontal gyrus, dorsolateral | 1 |
| left inferior frontal gyrus, triangular part | 1 |
| right superior occipital gyrus | 1 |
| left inferior occipital gyrus | 1 |
| right thalamus | 1 |
| right middle temporal gyrus | 1 |

| eTable 7. MDD without NSSI vs with NSSI（cov5） | |
| --- | --- |
| Regions | Number |
| Cerebelum_Crus1 | 4 |
| Vermis_4_5 | 4 |
| left Inferior frontal gyrus, opercular part | 3 |
| right amygdala | 3 |
| left cuneus | 2 |
| left thalamus | 2 |
| left superior temporal gyrus | 2 |
| left superior frontal gyrus, dorsolateral | 1 |
| left superior frontal gyrus, dorsolateral | 1 |
| right superior occipital gyrus | 1 |
| left inferior occipital gyrus | 1 |
| right thalamus | 1 |
| right middle temporal gyrus | 1 |
| left inferior temporal gyrus | 1 |
| Cerebelum_6 | 1 |

Part 2

Considering the impact of severe depression outcomes, we added the HAMD-17 score as a covariate based on the previous covariate setting to verify the differences of the above indicators.

1. Regional GMV differences

Compared with the MDD patients without a history of NSSI, the MDD patients with a history of NSSI showed significant GMV increases in the right superior temporal gyrus and right insula.

1. Regional ReHo differences

Compared with the MDD patients without a history of NSSI, the MDD patients with a history of NSSI showed a significant ReHo decrease in the right lingual gyrus.

The cluster size, peak T value, and peak MNI coordinates of regions with decreased GMV and Reho are listed in below.

| eTable 8. Regional GMV and ReHo differences | | | | | | |
| --- | --- | --- | --- | --- | --- | --- |
|  | region | cluster size | x | y | z | t |
| Reho | right lingual gyrus | 49 | 18 | -81 | -9 | 4.5777 |
| GMV | right superior temporal gyrus | 642 | 48 | -10.5 | -4.5 | 5.0423 |
|  | right insula | 104 |  | | | |
| MDD: major depressive disorder, NSSI: nonsuicidal self-injury, GMV: grey matter volume; ReHo: regional homogeneity analysis | | | | | | |

1. Global Connectome Topology

Across the defined threshold range, both patients and HCs demonstrated small-world topological properties (σ >1). At the large-scale network level, MDD patients with a history of NSSI showed significantly different sigma (*P*=0.009) compared with MDD patients without a history of NSSI (*P*=0.011). However, there were no significant differences in Eg (*P*=0.052), Lp (*P*=0.094) and Cp(*P*=0.719). No significant differences in BC, DC, NE were observed among the groups (*P* > 0.05, Bonferroni correction).

| eTable 9. MDD without NSSI vs with NSSI | | | | | | |
| --- | --- | --- | --- | --- | --- | --- |
|  | BC | | DC | | NE | |
| **Regions** | *p* | *t* | *p* | *t* | *p* | *t* |
| left precental gyrus | 7.60E-01 | -3.06E-01 | 5.86E-01 | 5.46E-01 | 9.81E-01 | 2.36E-02 |
| right precental gyrus | 8.62E-01 | 1.74E-01 | 1.45E-01 | 1.46E+00 | 4.12E-01 | 8.21E-01 |
| left superior frontal gyrus, dorsolateral | 2.43E-01 | 1.17E+00 | 9.52E-01 | -5.99E-02 | 7.57E-01 | -3.10E-01 |
| right superior frontal gyrus, dorsolateral | 3.30E-01 | 9.76E-01 | 9.97E-01 | 4.12E-03 | 7.52E-01 | -3.16E-01 |
| left superior frontal gyrus, orbital part | 5.91E-01 | -5.38E-01 | 1.54E-01 | -1.43E+00 | 1.03E-01 | -1.64E+00 |
| right superior frontal gyrus, orbital part | 3.07E-01 | -1.02E+00 | 1.64E-01 | -1.39E+00 | 7.99E-02 | -1.76E+00 |
| left middle frontal gyrus | 5.26E-01 | -6.36E-01 | 5.98E-01 | 5.27E-01 | 8.44E-01 | 1.97E-01 |
| right middle frontal gyrus | 5.96E-01 | -5.31E-01 | 6.52E-01 | 4.51E-01 | 9.89E-01 | 1.39E-02 |
| left middle frontal gyrus, orbital part | 8.92E-02 | 1.71E+00 | 8.95E-01 | 1.32E-01 | 6.19E-01 | -4.98E-01 |
| right middle frontal gyrus, orbital part | 9.07E-01 | -1.17E-01 | 2.92E-01 | -1.06E+00 | 2.46E-01 | -1.16E+00 |
| left inferior frontal gyrus, opercular part | 4.55E-01 | -7.49E-01 | 8.98E-01 | 1.29E-01 | 8.36E-01 | -2.08E-01 |
| right inferior frontal gyrus, opercular part | 3.15E-01 | 1.01E+00 | 6.02E-01 | -5.23E-01 | 3.83E-01 | -8.74E-01 |
| left inferior frontal gyrus, triangular part | 6.79E-01 | 4.14E-01 | 7.16E-01 | 3.64E-01 | 9.17E-01 | 1.04E-01 |
| right inferior frontal gyrus, triangular part | 5.90E-01 | -5.40E-01 | 5.18E-01 | -6.48E-01 | 3.42E-01 | -9.52E-01 |
| left inferior frontal gyrus, orbital part | 7.90E-01 | -2.67E-01 | 6.26E-02 | -1.87E+00 | 6.07E-02 | -1.89E+00 |
| right inferior frontal gyrus, orbital part | 2.37E-01 | 1.19E+00 | 6.06E-01 | -5.17E-01 | 4.62E-01 | -7.37E-01 |
| left rolandic operculum | 7.09E-01 | -3.73E-01 | 8.98E-01 | 1.29E-01 | 6.51E-01 | -4.53E-01 |
| right rolandic operculum | 3.98E-01 | -8.46E-01 | 9.99E-01 | -1.33E-03 | 4.15E-01 | -8.16E-01 |
| left supplementary motor area | 9.26E-01 | -9.27E-02 | 8.93E-01 | -1.35E-01 | 4.53E-01 | -7.52E-01 |
| right supplementary motor area | 9.30E-01 | -8.80E-02 | 9.53E-01 | -5.89E-02 | 3.34E-01 | -9.69E-01 |
| left olfactory cortex | 9.08E-01 | -1.16E-01 | 5.29E-01 | -6.31E-01 | 8.69E-01 | -1.65E-01 |
| right olfactory cortex | 4.06E-01 | -8.32E-01 | 7.87E-01 | -2.71E-01 | 8.55E-01 | -1.83E-01 |
| left superior frontal gyrus, medial | 3.26E-01 | 9.84E-01 | 3.08E-01 | -1.02E+00 | 2.17E-01 | -1.24E+00 |
| right superior frontal gyrus, medial | 3.37E-01 | 9.62E-01 | 6.96E-01 | -3.91E-01 | 4.22E-01 | -8.04E-01 |
| left superior frontal gyrus, medial orbital | 3.20E-01 | 9.97E-01 | 2.24E-01 | -1.22E+00 | 1.01E-01 | -1.65E+00 |
| right superior frontal gyrus, medial orbital | 4.75E-01 | 7.16E-01 | 3.55E-01 | -9.27E-01 | 1.20E-01 | -1.56E+00 |
| left gyrus rectus | 1.22E-01 | -1.55E+00 | 3.86E-03 | -2.92E+00 | 2.73E-03 | -3.03E+00 |
| right gyrus rectus | 1.39E-01 | -1.48E+00 | 2.74E-02 | -2.22E+00 | 1.17E-02 | -2.54E+00 |
| left insula | 7.25E-01 | 3.52E-01 | 6.37E-01 | -4.73E-01 | 4.82E-01 | -7.05E-01 |
| right insula | 9.11E-01 | -1.11E-01 | 6.93E-01 | -3.96E-01 | 2.97E-01 | -1.05E+00 |
| left anterior cingulate and paracingulate gyri | 6.44E-01 | 4.63E-01 | 4.40E-01 | -7.73E-01 | 2.74E-01 | -1.10E+00 |
| right anterior cingulate and paracingulate gyri | 4.50E-01 | 7.57E-01 | 5.36E-01 | -6.20E-01 | 3.84E-01 | -8.72E-01 |
| left median cingulate and paracingulate gyri | 1.43E-01 | 1.47E+00 | 5.24E-02 | 1.95E+00 | 1.10E-01 | 1.60E+00 |
| right median cingulate and paracingulate gyri | 5.55E-01 | 5.91E-01 | 1.95E-01 | 1.30E+00 | 4.90E-01 | 6.91E-01 |
| left posterior cingulate gyrus | 7.63E-02 | -1.78E+00 | 3.47E-01 | -9.42E-01 | 1.06E-01 | -1.62E+00 |
| right posterior cingulate gyrus | 7.37E-01 | 3.37E-01 | 9.01E-01 | 1.24E-01 | 3.85E-01 | -8.70E-01 |
| left hippocampus | 6.58E-01 | 4.44E-01 | 8.17E-01 | -2.32E-01 | 7.05E-01 | -3.79E-01 |
| right hippocampus | 1.18E-01 | -1.57E+00 | 1.52E-01 | -1.44E+00 | 8.58E-02 | -1.73E+00 |
| left parahippocampal gyrus | 4.67E-01 | -7.28E-01 | 4.07E-01 | -8.31E-01 | 2.79E-01 | -1.09E+00 |
| right parahippocampal gyrus | 5.13E-01 | -6.55E-01 | 4.30E-01 | -7.91E-01 | 2.79E-01 | -1.08E+00 |
| left amygdala | 7.16E-01 | -3.64E-01 | 9.35E-01 | 8.14E-02 | 6.20E-01 | -4.97E-01 |
| right amygdala | 4.07E-01 | 8.30E-01 | 7.38E-01 | 3.35E-01 | 5.55E-01 | -5.91E-01 |
| left calcarine fissure and surrounding cortex | 9.61E-01 | -4.89E-02 | 7.16E-01 | 3.65E-01 | 8.88E-01 | -1.42E-01 |
| right calcarine fissure and surrounding cortex | 5.38E-01 | -6.16E-01 | 9.98E-01 | 2.51E-03 | 5.64E-01 | -5.77E-01 |
| left cuneus | 8.71E-01 | 1.63E-01 | 7.65E-01 | 2.99E-01 | 9.98E-01 | -2.82E-03 |
| right cuneus | 7.80E-01 | 2.80E-01 | 9.97E-01 | 3.68E-03 | 7.15E-01 | -3.65E-01 |
| left lingual gyrus | 6.27E-01 | 4.86E-01 | 4.42E-01 | 7.70E-01 | 8.28E-01 | 2.18E-01 |
| right lingual gyrus | 5.67E-01 | 5.73E-01 | 5.13E-01 | 6.55E-01 | 9.50E-01 | 6.23E-02 |
| left superior occipital gyrus | 5.07E-01 | 6.65E-01 | 2.22E-01 | 1.22E+00 | 3.50E-01 | 9.37E-01 |
| right superior occipital gyrus | 8.63E-01 | 1.73E-01 | 9.61E-01 | 4.84E-02 | 9.06E-01 | -1.18E-01 |
| left middle occipital gyrus | 5.87E-01 | 5.44E-01 | 3.90E-01 | 8.61E-01 | 5.75E-01 | 5.62E-01 |
| right middle occipital gyrus | 8.64E-01 | 1.71E-01 | 6.02E-01 | 5.22E-01 | 9.84E-01 | -2.03E-02 |
| left inferior occipital gyrus | 4.77E-01 | -7.13E-01 | 4.22E-01 | 8.05E-01 | 7.74E-01 | 2.87E-01 |
| right inferior occipital gyrus | 2.13E-01 | -1.25E+00 | 9.86E-01 | -1.71E-02 | 6.50E-01 | -4.54E-01 |
| left fusiform gyrus | 1.61E-01 | 1.41E+00 | 1.76E-01 | 1.36E+00 | 5.69E-01 | 5.70E-01 |
| right fusiform gyrus | 6.98E-02 | -1.82E+00 | 2.94E-01 | -1.05E+00 | 9.50E-02 | -1.68E+00 |
| left postcentral gyrus | 3.08E-01 | -1.02E+00 | 4.21E-01 | 8.06E-01 | 7.32E-01 | 3.43E-01 |
| right postcentral gyrus | 6.49E-01 | -4.56E-01 | 7.62E-01 | 3.04E-01 | 7.49E-01 | -3.21E-01 |
| left superior parietal gyrus | 6.76E-01 | -4.18E-01 | 4.01E-01 | 8.41E-01 | 7.53E-01 | 3.15E-01 |
| right superior parietal gyrus | 8.73E-02 | -1.72E+00 | 2.89E-01 | -1.06E+00 | 6.54E-02 | -1.85E+00 |
| left inferior parietal, but supramarginal and angular gyri | 1.73E-01 | 1.37E+00 | 1.87E-02 | 2.37E+00 | 4.66E-02 | 2.00E+00 |
| right inferior parietal, but supramarginal and angular gyri | 8.46E-01 | 1.95E-01 | 9.03E-01 | -1.22E-01 | 8.35E-01 | -2.09E-01 |
| left supramarginal gyrus | 2.74E-01 | 1.10E+00 | 2.34E-01 | 1.19E+00 | 3.41E-01 | 9.55E-01 |
| right supramarginal gyrus | 4.10E-01 | -8.25E-01 | 7.98E-01 | -2.56E-01 | 3.92E-01 | -8.58E-01 |
| left angular gyrus | 4.56E-01 | 7.46E-01 | 2.06E-02 | 2.33E+00 | 9.93E-02 | 1.66E+00 |
| right angular gyrus | 8.19E-01 | 2.29E-01 | 9.11E-01 | 1.12E-01 | 9.89E-01 | 1.38E-02 |
| left precuneus | 8.35E-02 | -1.74E+00 | 6.36E-01 | -4.73E-01 | 4.45E-01 | -7.64E-01 |
| right precuneus | 1.41E-01 | -1.48E+00 | 3.85E-01 | -8.70E-01 | 1.85E-01 | -1.33E+00 |
| left paracentral lobule | 4.88E-01 | -6.94E-01 | 6.10E-01 | 5.10E-01 | 9.56E-01 | 5.55E-02 |
| right paracentral lobule | 2.17E-01 | -1.24E+00 | 8.10E-01 | -2.41E-01 | 3.31E-01 | -9.73E-01 |
| left caudate nucleus | 4.58E-02 | -2.01E+00 | 6.05E-01 | -5.17E-01 | 6.80E-01 | -4.13E-01 |
| right caudate nucleus | 9.25E-01 | 9.44E-02 | 7.40E-01 | 3.33E-01 | 9.78E-01 | -2.76E-02 |
| left lenticular nucleus, putamen | 5.41E-01 | 6.13E-01 | 4.53E-01 | 7.52E-01 | 7.57E-01 | 3.10E-01 |
| right lenticular nucleus, putamen | 2.43E-02 | -2.27E+00 | 3.77E-01 | -8.86E-01 | 2.62E-01 | -1.12E+00 |
| left lenticular nucleus, pallidum | 1.03E-01 | -1.63E+00 | 6.45E-01 | -4.62E-01 | 8.11E-01 | -2.39E-01 |
| right lenticular nucleus, pallidum | 2.87E-03 | -3.01E+00 | 3.02E-01 | -1.03E+00 | 3.29E-01 | -9.79E-01 |
| left thalamus | 9.78E-02 | 1.66E+00 | 1.63E-01 | 1.40E+00 | 3.99E-01 | 8.46E-01 |
| right thalamus | 5.02E-02 | 1.97E+00 | 3.73E-01 | 8.93E-01 | 6.31E-01 | 4.82E-01 |
| left heschl gyrus | 7.04E-02 | -1.82E+00 | 1.92E-01 | -1.31E+00 | 7.45E-02 | -1.79E+00 |
| right heschl gyrus | 8.16E-04 | -3.39E+00 | 1.80E-02 | -2.38E+00 | 1.55E-02 | -2.44E+00 |
| left superior temporal gyrus | 9.39E-01 | 7.64E-02 | 1.29E-01 | 1.52E+00 | 3.23E-01 | 9.90E-01 |
| right superior temporal gyrus | 6.14E-01 | 5.05E-01 | 6.01E-01 | 5.24E-01 | 9.92E-01 | 1.04E-02 |
| left temporal pole: superior temporal gyrus | 9.27E-01 | 9.15E-02 | 8.30E-01 | 2.14E-01 | 7.26E-01 | -3.51E-01 |
| right temporal pole: superior temporal gyrus | 9.28E-01 | 9.04E-02 | 7.71E-01 | 2.92E-01 | 8.07E-01 | -2.44E-01 |
| left middle temporal gyrus | 3.23E-01 | 9.89E-01 | 3.23E-02 | 2.15E+00 | 5.74E-02 | 1.91E+00 |
| right middle temporal gyrus | 3.16E-01 | 1.01E+00 | 5.89E-01 | 5.41E-01 | 7.03E-01 | 3.82E-01 |
| left temporal pole: middle temporal gyrus | 9.70E-01 | 3.75E-02 | 6.34E-01 | -4.77E-01 | 2.95E-01 | -1.05E+00 |
| right temporal pole: middle temporal gyrus | 3.85E-01 | -8.70E-01 | 1.48E-01 | -1.45E+00 | 8.34E-02 | -1.74E+00 |
| left inferior temporal gyrus | 6.05E-01 | 5.18E-01 | 2.37E-01 | 1.18E+00 | 3.90E-01 | 8.62E-01 |
| right inferior temporal gyrus | 2.89E-01 | -1.06E+00 | 9.54E-01 | -5.76E-02 | 7.10E-01 | -3.73E-01 |
| Cerebelum_Crus1_L | 9.17E-01 | 1.05E-01 | 1.18E-01 | 1.57E+00 | 3.21E-01 | 9.94E-01 |
| Cerebelum_Crus1_R | 7.31E-01 | 3.45E-01 | 4.61E-01 | 7.38E-01 | 7.96E-01 | 2.59E-01 |
| Cerebelum_Crus2_L | 4.68E-01 | -7.27E-01 | 7.73E-01 | 2.88E-01 | 8.68E-01 | 1.67E-01 |
| Cerebelum_Crus2_R | 4.26E-02 | 2.04E+00 | 2.18E-01 | 1.24E+00 | 3.95E-01 | 8.53E-01 |
| Cerebelum_3_L | 7.03E-01 | 3.81E-01 | 8.31E-01 | -2.14E-01 | 4.80E-01 | -7.08E-01 |
| Cerebelum_3_R | 4.36E-01 | -7.81E-01 | 2.24E-01 | -1.22E+00 | 2.64E-01 | -1.12E+00 |
| Cerebelum_4_5_L | 3.70E-01 | 8.98E-01 | 6.31E-01 | 4.80E-01 | 8.73E-01 | 1.60E-01 |
| Cerebelum_4_5_R | 3.27E-01 | 9.81E-01 | 8.83E-01 | 1.47E-01 | 8.56E-01 | -1.82E-01 |
| Cerebelum_6_L | 9.67E-01 | -4.16E-02 | 1.04E-01 | 1.63E+00 | 3.08E-01 | 1.02E+00 |
| Cerebelum_6_R | 1.16E-01 | -1.58E+00 | 7.28E-01 | 3.49E-01 | 9.18E-01 | 1.03E-01 |
| Cerebelum_7b_L | 3.40E-01 | -9.56E-01 | 8.40E-01 | -2.02E-01 | 9.50E-01 | -6.27E-02 |
| Cerebelum_7b_R | 9.53E-01 | 5.96E-02 | 8.18E-01 | -2.30E-01 | 4.76E-01 | -7.14E-01 |
| Cerebelum_8_L | 2.30E-01 | -1.20E+00 | 7.00E-01 | 3.86E-01 | 7.52E-01 | 3.16E-01 |
| Cerebelum_8_R | 3.96E-01 | -8.51E-01 | 8.86E-01 | -1.43E-01 | 5.22E-01 | -6.41E-01 |
| Cerebelum_9_L | 2.34E-01 | -1.19E+00 | 9.20E-01 | 1.00E-01 | 8.07E-01 | -2.44E-01 |
| Cerebelum_9_R | 2.23E-01 | -1.22E+00 | 8.20E-01 | -2.28E-01 | 6.69E-01 | -4.28E-01 |
| Cerebelum_10_L | 9.90E-01 | 1.21E-02 | 3.53E-01 | -9.31E-01 | 4.70E-02 | -2.00E+00 |
| Cerebelum_10_R | 1.07E-01 | -1.62E+00 | 3.90E-01 | -8.61E-01 | 2.05E-01 | -1.27E+00 |
| Vermis_1_2 | 2.10E-01 | -1.26E+00 | 1.32E-01 | -1.51E+00 | 3.75E-02 | -2.09E+00 |
| Vermis_3 | 8.89E-01 | 1.40E-01 | 4.66E-01 | -7.30E-01 | 3.88E-01 | -8.65E-01 |
| Vermis_4_5 | 2.93E-01 | -1.05E+00 | 6.40E-01 | -4.69E-01 | 1.75E-01 | -1.36E+00 |
| Vermis_6 | 4.26E-01 | 7.97E-01 | 2.74E-02 | 2.22E+00 | 1.85E-01 | 1.33E+00 |
| Vermis_7 | 4.88E-01 | 6.94E-01 | 6.88E-01 | 4.03E-01 | 8.57E-01 | 1.80E-01 |
| Vermis_8 | 4.51E-01 | -7.54E-01 | 6.23E-01 | -4.92E-01 | 5.66E-01 | -5.74E-01 |
| Vermis_9 | 4.66E-01 | 7.30E-01 | 8.54E-01 | 1.85E-01 | 9.66E-01 | 4.23E-02 |
| Vermis_10 | 8.42E-01 | 2.00E-01 | 8.63E-01 | -1.73E-01 | 1.42E-01 | -1.47E+00 |
| DC: Degree centrality, BC: Betweenness centrality, NE: local efficiency | | | | | | |

Compared with the MDD patients without NSSI history, the MDD patients with NSSI history showed significant decreases network in cerebellum-centre network(16 edges, 15 nodes), including important nodes in the cerebellum (left cerebellum_superior 4 edges, Vermis_6 4 edges), the left inferior frontal gyrus opercular part (3 edges), the right amygdala (3 edges), and the left thalamus (3 edges).

| eTable 10. MDD without NSSI vs with NSSI | | |
| --- | --- | --- |
| node | node | number |
| 91 | Cerebelum_Crus1_L | 4 |
| 112 | Vermis_6 | 4 |
| 11 | Left Inferior frontal gyrus, opercular part | 3 |
| 42 | Right amygdala | 3 |
| 45 | Left Cuneus | 2 |
| 77 | Left Thalamus | 2 |
| 81 | Left Superior temporal gyrus | 2 |
| 3 | Left superior frontal gyrus, dorsolateral | 1 |
| 23 | Left superior frontal gyrus, dorsolateral | 1 |
| 50 | Right superior occipital gyrus | 1 |
| 53 | Left superior occipital gyrus | 1 |
| 78 | Right thalamus | 1 |
| 86 | Right middle temporal gyrus | 1 |
| 89 | Left inferior temporal gyrus | 1 |
| 99 | Cerebelum_6_L | 1 |

Part 3

Considering the age-range broad, we made more restrictive age-inclusion include participant younger than 30 years old. A total of 73 MDD patients with NSSI history and 124 MDD patients without NSSI history were retained for analysis, with age, sex, depression course, education level and HAMD-17 scores as nuisance covariates.

1. Regional GMV differences

Compared with the MDD patients without a history of NSSI, the MDD patients with a history of NSSI showed significant GMV increases in the right superior temporal gyrus, right insula and left inferior frontal gyrus triangular part (with a voxelwise *P value* of 0.001 and a Gaussian random field corrected clusterwise P *value* of 0.05).

1. Regional ReHo differences

Compared with the MDD patients without a history of NSSI, the MDD patients with a history of NSSI showed a significant ReHo decrease in the right lingual gyrus (with a voxelwise *P value* of 0.001 and a FWE corrected clusterwise P *value* of 0.05).

The cluster size, peak T value, and peak MNI coordinates of regions with decreased GMV and Reho are listed in below.

| eTable 11. Regional GMV and ReHo differences | | | | | | |
| --- | --- | --- | --- | --- | --- | --- |
|  | region | cluster size | x | y | z | t |
| Reho | right lingual gyrus | 31 | 18 | -81 | -9 | 4.7852 |
| GMV | right superior temporal gyrus | 477 | 49.5 | -9 | -6 | 4.7151 |
|  | right insula | 71 |  | | | |
|  | left inferior frontal gyrus triangular part | 256 | -54 | 33 | 3 | 4.0163 |
| MDD: major depressive disorder, NSSI: nonsuicidal self-injury, GMV: grey matter volume; ReHo: regional homogeneity analysis | | | | | | |

1. Global Connectome Topology

Across the defined threshold range, both patients and HCs demonstrated small-world topological properties (σ >1). At the large-scale network level, MDD patients with a history of NSSI showed significantly different sigma (P=0.009) and Eg (P=0.022) compared with MDD patients without a history of NSSI. However, there were no significant differences in Lp (P=0.055) and Cp(P=0.482). No significant differences in BC, DC, NE were observed among the groups (P > 0.05, Bonferroni correction).

| eTable 12. MDD without NSSI vs with NSSI | | | | | | |
| --- | --- | --- | --- | --- | --- | --- |
|  | BC | | DC | | NE | |
| **Regions** | *p* | *t* | *p* | *t* | *p* | *t* |
| left precental gyrus | 5.67E-01 | 5.73E-01 | 4.32E-01 | -7.87E-01 | 8.67E-01 | -1.68E-01 |
| right precental gyrus | 6.06E-01 | 5.16E-01 | 3.39E-01 | -9.58E-01 | 7.94E-01 | -2.62E-01 |
| left superior frontal gyrus, dorsolateral | 1.32E-01 | -1.51E+00 | 9.48E-01 | -6.58E-02 | 7.70E-01 | 2.93E-01 |
| right superior frontal gyrus, dorsolateral | 5.30E-01 | -6.29E-01 | 7.64E-01 | 3.01E-01 | 4.58E-01 | 7.43E-01 |
| left superior frontal gyrus, orbital part | 5.87E-01 | 5.44E-01 | 1.50E-01 | 1.45E+00 | 1.11E-01 | 1.60E+00 |
| right superior frontal gyrus, orbital part | 2.94E-01 | 1.05E+00 | 2.29E-01 | 1.21E+00 | 1.23E-01 | 1.55E+00 |
| left middle frontal gyrus | 4.86E-01 | 6.98E-01 | 5.33E-01 | -6.25E-01 | 7.84E-01 | -2.75E-01 |
| right middle frontal gyrus | 2.21E-01 | 1.23E+00 | 9.18E-01 | -1.03E-01 | 6.43E-01 | 4.65E-01 |
| left middle frontal gyrus, orbital part | 1.60E-01 | -1.41E+00 | 7.73E-01 | -2.89E-01 | 7.85E-01 | 2.73E-01 |
| right middle frontal gyrus, orbital part | 6.56E-01 | 4.46E-01 | 3.41E-01 | 9.54E-01 | 3.10E-01 | 1.02E+00 |
| left inferior frontal gyrus, opercular part | 7.87E-01 | 2.71E-01 | 4.32E-01 | -7.87E-01 | 7.62E-01 | -3.03E-01 |
| right inferior frontal gyrus, opercular part | 4.44E-01 | -7.67E-01 | 4.04E-01 | 8.36E-01 | 2.09E-01 | 1.26E+00 |
| left inferior frontal gyrus, triangular part | 7.03E-01 | -3.82E-01 | 3.86E-01 | -8.69E-01 | 5.64E-01 | -5.78E-01 |
| right inferior frontal gyrus, triangular part | 4.78E-01 | 7.10E-01 | 3.43E-01 | 9.50E-01 | 1.73E-01 | 1.37E+00 |
| left inferior frontal gyrus, orbital part | 4.03E-01 | -8.39E-01 | 2.98E-01 | 1.04E+00 | 2.53E-01 | 1.15E+00 |
| right inferior frontal gyrus, orbital part | 4.08E-01 | -8.30E-01 | 5.81E-01 | 5.53E-01 | 4.09E-01 | 8.27E-01 |
| left rolandic operculum | 9.28E-01 | 9.11E-02 | 4.41E-01 | -7.72E-01 | 9.74E-01 | -3.20E-02 |
| right rolandic operculum | 3.42E-01 | 9.52E-01 | 8.92E-01 | -1.36E-01 | 4.16E-01 | 8.14E-01 |
| left supplementary motor area | 7.43E-01 | -3.28E-01 | 5.62E-01 | -5.81E-01 | 9.21E-01 | 9.96E-02 |
| right supplementary motor area | 7.38E-01 | 3.34E-01 | 9.37E-01 | -7.92E-02 | 3.75E-01 | 8.88E-01 |
| left olfactory cortex | 6.88E-01 | 4.03E-01 | 2.64E-01 | 1.12E+00 | 4.69E-01 | 7.25E-01 |
| right olfactory cortex | 5.71E-01 | 5.68E-01 | 6.02E-01 | 5.22E-01 | 6.42E-01 | 4.65E-01 |
| left superior frontal gyrus, medial | 2.23E-01 | -1.22E+00 | 3.73E-01 | 8.93E-01 | 2.24E-01 | 1.22E+00 |
| right superior frontal gyrus, medial | 3.85E-01 | -8.70E-01 | 7.03E-01 | 3.81E-01 | 3.79E-01 | 8.81E-01 |
| left superior frontal gyrus, medial orbital | 2.99E-01 | -1.04E+00 | 3.17E-01 | 1.00E+00 | 1.47E-01 | 1.45E+00 |
| right superior frontal gyrus, medial orbital | 2.44E-01 | -1.17E+00 | 6.03E-01 | 5.21E-01 | 2.13E-01 | 1.25E+00 |
| left gyrus rectus | 6.47E-02 | 1.86E+00 | 3.97E-03 | 2.92E+00 | 2.34E-03 | 3.08E+00 |
| right gyrus rectus | 2.82E-01 | 1.08E+00 | 5.93E-02 | 1.90E+00 | 2.25E-02 | 2.30E+00 |
| left insula | 9.04E-01 | 1.21E-01 | 7.85E-01 | 2.73E-01 | 5.70E-01 | 5.69E-01 |
| right insula | 6.22E-01 | 4.94E-01 | 5.48E-01 | 6.02E-01 | 1.91E-01 | 1.31E+00 |
| left anterior cingulate and paracingulate gyri | 8.57E-01 | -1.80E-01 | 4.87E-01 | 6.96E-01 | 3.00E-01 | 1.04E+00 |
| right anterior cingulate and paracingulate gyri | 7.47E-01 | -3.23E-01 | 4.37E-01 | 7.78E-01 | 2.97E-01 | 1.05E+00 |
| left median cingulate and paracingulate gyri | 3.31E-01 | -9.75E-01 | 1.48E-01 | -1.45E+00 | 3.00E-01 | -1.04E+00 |
| right median cingulate and paracingulate gyri | 8.90E-01 | -1.39E-01 | 2.81E-01 | -1.08E+00 | 6.79E-01 | -4.14E-01 |
| left posterior cingulate gyrus | 6.48E-02 | 1.86E+00 | 1.87E-01 | 1.32E+00 | 4.87E-02 | 1.98E+00 |
| right posterior cingulate gyrus | 6.13E-01 | -5.07E-01 | 8.85E-01 | -1.45E-01 | 3.37E-01 | 9.63E-01 |
| left hippocampus | 4.21E-01 | -8.07E-01 | 8.36E-01 | -2.08E-01 | 9.57E-01 | 5.40E-02 |
| right hippocampus | 8.73E-02 | 1.72E+00 | 9.36E-02 | 1.68E+00 | 5.30E-02 | 1.95E+00 |
| left parahippocampal gyrus | 9.47E-01 | -6.61E-02 | 6.61E-01 | 4.39E-01 | 3.60E-01 | 9.18E-01 |
| right parahippocampal gyrus | 4.89E-01 | 6.93E-01 | 5.09E-01 | 6.61E-01 | 3.18E-01 | 1.00E+00 |
| left amygdala | 6.42E-01 | 4.66E-01 | 7.46E-01 | 3.24E-01 | 2.75E-01 | 1.09E+00 |
| right amygdala | 5.99E-01 | -5.27E-01 | 9.06E-01 | -1.19E-01 | 3.60E-01 | 9.17E-01 |
| left calcarine fissure and surrounding cortex | 6.72E-01 | 4.24E-01 | 8.94E-01 | 1.33E-01 | 5.18E-01 | 6.48E-01 |
| right calcarine fissure and surrounding cortex | 2.72E-01 | 1.10E+00 | 7.15E-01 | 3.66E-01 | 3.11E-01 | 1.02E+00 |
| left cuneus | 9.03E-01 | 1.22E-01 | 9.39E-01 | 7.71E-02 | 6.35E-01 | 4.75E-01 |
| right cuneus | 8.08E-01 | -2.44E-01 | 8.06E-01 | 2.46E-01 | 4.58E-01 | 7.44E-01 |
| left lingual gyrus | 6.52E-01 | -4.52E-01 | 3.85E-01 | -8.72E-01 | 7.79E-01 | -2.82E-01 |
| right lingual gyrus | 4.94E-01 | -6.85E-01 | 3.53E-01 | -9.30E-01 | 7.22E-01 | -3.56E-01 |
| left superior occipital gyrus | 7.02E-01 | -3.83E-01 | 3.56E-01 | -9.25E-01 | 5.72E-01 | -5.65E-01 |
| right superior occipital gyrus | 9.71E-01 | 3.68E-02 | 7.89E-01 | 2.67E-01 | 6.52E-01 | 4.52E-01 |
| left middle occipital gyrus | 9.10E-01 | 1.13E-01 | 8.94E-01 | -1.33E-01 | 7.49E-01 | 3.20E-01 |
| right middle occipital gyrus | 7.97E-01 | 2.57E-01 | 8.97E-01 | -1.30E-01 | 6.57E-01 | 4.45E-01 |
| left inferior occipital gyrus | 4.09E-01 | 8.27E-01 | 5.05E-01 | -6.68E-01 | 9.44E-01 | -6.99E-02 |
| right inferior occipital gyrus | 2.74E-01 | 1.10E+00 | 9.55E-01 | 5.62E-02 | 6.64E-01 | 4.36E-01 |
| left fusiform gyrus | 2.18E-01 | -1.24E+00 | 3.56E-01 | -9.26E-01 | 9.41E-01 | -7.35E-02 |
| right fusiform gyrus | 2.84E-01 | 1.08E+00 | 3.64E-01 | 9.11E-01 | 1.13E-01 | 1.59E+00 |
| left postcentral gyrus | 1.34E-01 | 1.51E+00 | 4.19E-01 | -8.09E-01 | 7.89E-01 | -2.68E-01 |
| right postcentral gyrus | 4.35E-01 | 7.83E-01 | 8.63E-01 | -1.73E-01 | 6.05E-01 | 5.19E-01 |
| left superior parietal gyrus | 5.01E-01 | 6.74E-01 | 5.54E-01 | -5.93E-01 | 9.96E-01 | -4.93E-03 |
| right superior parietal gyrus | 6.61E-02 | 1.85E+00 | 2.17E-01 | 1.24E+00 | 3.65E-02 | 2.11E+00 |
| left inferior parietal, but supramarginal and angular gyri | 1.17E-01 | -1.57E+00 | 1.59E-02 | -2.43E+00 | 5.41E-02 | -1.94E+00 |
| right inferior parietal, but supramarginal and angular gyri | 8.01E-01 | -2.53E-01 | 9.80E-01 | 2.45E-02 | 8.40E-01 | 2.02E-01 |
| left supramarginal gyrus | 3.47E-01 | -9.42E-01 | 3.46E-01 | -9.45E-01 | 7.12E-01 | -3.70E-01 |
| right supramarginal gyrus | 3.70E-01 | 8.99E-01 | 7.17E-01 | 3.63E-01 | 2.67E-01 | 1.11E+00 |
| left angular gyrus | 6.67E-01 | -4.31E-01 | 5.04E-02 | -1.97E+00 | 1.21E-01 | -1.56E+00 |
| right angular gyrus | 9.88E-01 | 1.45E-02 | 9.06E-01 | 1.18E-01 | 8.27E-01 | 2.19E-01 |
| left precuneus | 2.63E-02 | 2.24E+00 | 3.75E-01 | 8.89E-01 | 1.98E-01 | 1.29E+00 |
| right precuneus | 1.17E-01 | 1.58E+00 | 2.15E-01 | 1.24E+00 | 7.07E-02 | 1.82E+00 |
| left paracentral lobule | 5.59E-01 | 5.86E-01 | 4.53E-01 | -7.52E-01 | 7.81E-01 | -2.79E-01 |
| right paracentral lobule | 2.42E-01 | 1.17E+00 | 8.44E-01 | 1.97E-01 | 3.37E-01 | 9.63E-01 |
| left caudate nucleus | 9.44E-02 | 1.68E+00 | 3.82E-01 | 8.77E-01 | 4.86E-01 | 6.98E-01 |
| right caudate nucleus | 5.97E-01 | 5.30E-01 | 8.55E-01 | 1.83E-01 | 6.10E-01 | 5.12E-01 |
| left lenticular nucleus, putamen | 6.85E-01 | -4.06E-01 | 5.08E-01 | -6.63E-01 | 9.49E-01 | -6.41E-02 |
| right lenticular nucleus, putamen | 1.65E-02 | 2.42E+00 | 2.89E-01 | 1.06E+00 | 1.31E-01 | 1.52E+00 |
| left lenticular nucleus, pallidum | 1.02E-01 | 1.64E+00 | 3.92E-01 | 8.58E-01 | 4.09E-01 | 8.27E-01 |
| right lenticular nucleus, pallidum | 2.79E-03 | 3.03E+00 | 2.29E-01 | 1.21E+00 | 1.72E-01 | 1.37E+00 |
| left thalamus | 1.35E-01 | -1.50E+00 | 1.17E-01 | -1.57E+00 | 4.20E-01 | -8.08E-01 |
| right thalamus | 5.83E-02 | -1.90E+00 | 3.83E-01 | -8.74E-01 | 6.67E-01 | -4.31E-01 |
| left heschl gyrus | 1.85E-01 | 1.33E+00 | 6.25E-01 | 4.89E-01 | 2.10E-01 | 1.26E+00 |
| right heschl gyrus | 8.28E-04 | 3.40E+00 | 5.42E-02 | 1.94E+00 | 3.55E-02 | 2.12E+00 |
| left superior temporal gyrus | 9.94E-01 | 7.41E-03 | 5.80E-02 | -1.91E+00 | 2.14E-01 | -1.25E+00 |
| right superior temporal gyrus | 8.29E-01 | -2.17E-01 | 5.15E-01 | -6.53E-01 | 9.70E-01 | -3.80E-02 |
| left temporal pole: superior temporal gyrus | 6.59E-01 | -4.42E-01 | 4.39E-01 | -7.75E-01 | 9.30E-01 | -8.84E-02 |
| right temporal pole: superior temporal gyrus | 7.98E-01 | 2.56E-01 | 6.52E-01 | -4.52E-01 | 9.05E-01 | 1.20E-01 |
| left middle temporal gyrus | 2.78E-01 | -1.09E+00 | 4.87E-02 | -1.98E+00 | 1.07E-01 | -1.62E+00 |
| right middle temporal gyrus | 3.46E-01 | -9.44E-01 | 6.40E-01 | -4.68E-01 | 8.90E-01 | -1.38E-01 |
| left temporal pole: middle temporal gyrus | 9.44E-01 | 7.05E-02 | 5.06E-01 | 6.66E-01 | 2.18E-01 | 1.23E+00 |
| right temporal pole: middle temporal gyrus | 4.84E-01 | 7.01E-01 | 1.38E-01 | 1.49E+00 | 7.00E-02 | 1.82E+00 |
| left inferior temporal gyrus | 4.99E-01 | -6.78E-01 | 2.89E-01 | -1.06E+00 | 5.09E-01 | -6.62E-01 |
| right inferior temporal gyrus | 7.40E-01 | 3.32E-01 | 8.18E-01 | -2.31E-01 | 8.93E-01 | 1.35E-01 |
| Cerebelum_Crus1_L | 9.38E-01 | -7.85E-02 | 1.76E-01 | -1.36E+00 | 4.46E-01 | -7.63E-01 |
| Cerebelum_Crus1_R | 5.55E-01 | -5.91E-01 | 4.64E-01 | -7.34E-01 | 8.18E-01 | -2.30E-01 |
| Cerebelum_Crus2_L | 4.13E-01 | 8.20E-01 | 8.56E-01 | 1.81E-01 | 7.54E-01 | 3.14E-01 |
| Cerebelum_Crus2_R | 5.77E-02 | -1.91E+00 | 2.99E-01 | -1.04E+00 | 5.22E-01 | -6.42E-01 |
| Cerebelum_3_L | 5.77E-01 | -5.59E-01 | 8.44E-01 | 1.97E-01 | 4.49E-01 | 7.58E-01 |
| Cerebelum_3_R | 3.67E-01 | 9.04E-01 | 1.63E-01 | 1.40E+00 | 1.29E-01 | 1.52E+00 |
| Cerebelum_4_5_L | 7.71E-01 | -2.91E-01 | 5.49E-01 | -6.00E-01 | 8.12E-01 | -2.38E-01 |
| Cerebelum_4_5_R | 1.38E-01 | -1.49E+00 | 3.92E-01 | -8.58E-01 | 6.29E-01 | -4.84E-01 |
| Cerebelum_6_L | 9.48E-01 | 6.55E-02 | 6.61E-02 | -1.85E+00 | 2.64E-01 | -1.12E+00 |
| Cerebelum_6_R | 1.97E-01 | 1.29E+00 | 4.12E-01 | -8.22E-01 | 6.29E-01 | -4.84E-01 |
| Cerebelum_7b_L | 2.69E-01 | 1.11E+00 | 7.47E-01 | 3.23E-01 | 8.77E-01 | 1.55E-01 |
| Cerebelum_7b_R | 4.34E-01 | -7.84E-01 | 8.99E-01 | -1.27E-01 | 6.53E-01 | 4.50E-01 |
| Cerebelum_8_L | 2.24E-01 | 1.22E+00 | 6.56E-01 | -4.45E-01 | 7.70E-01 | -2.93E-01 |
| Cerebelum_8_R | 8.67E-01 | -1.68E-01 | 5.38E-01 | -6.18E-01 | 9.77E-01 | 2.86E-02 |
| Cerebelum_9_L | 9.94E-02 | 1.66E+00 | 6.83E-01 | 4.09E-01 | 3.48E-01 | 9.41E-01 |
| Cerebelum_9_R | 3.67E-01 | 9.04E-01 | 6.58E-01 | 4.43E-01 | 4.48E-01 | 7.60E-01 |
| Cerebelum_10_L | 7.73E-01 | 2.89E-01 | 5.82E-01 | 5.51E-01 | 5.78E-02 | 1.91E+00 |
| Cerebelum_10_R | 1.54E-01 | 1.43E+00 | 9.82E-01 | -2.31E-02 | 5.56E-01 | 5.89E-01 |
| Vermis_1_2 | 5.78E-02 | 1.91E+00 | 6.36E-02 | 1.87E+00 | 1.27E-02 | 2.52E+00 |
| Vermis_3 | 9.74E-01 | 3.31E-02 | 4.25E-01 | 7.99E-01 | 3.60E-01 | 9.17E-01 |
| Vermis_4_5 | 5.90E-01 | 5.40E-01 | 9.51E-01 | 6.09E-02 | 2.96E-01 | 1.05E+00 |
| Vermis_6 | 7.71E-01 | -2.91E-01 | 3.76E-02 | -2.09E+00 | 2.44E-01 | -1.17E+00 |
| Vermis_7 | 8.93E-01 | 1.35E-01 | 9.33E-01 | -8.36E-02 | 9.62E-01 | 4.77E-02 |
| Vermis_8 | 4.85E-01 | 7.00E-01 | 4.68E-01 | 7.27E-01 | 4.92E-01 | 6.88E-01 |
| Vermis_9 | 7.72E-01 | -2.90E-01 | 8.39E-01 | 2.04E-01 | 6.66E-01 | 4.32E-01 |
| Vermis_10 | 7.73E-01 | 2.89E-01 | 9.52E-01 | -6.07E-02 | 8.96E-02 | 1.71E+00 |
| DC: Degree centrality, BC: Betweenness centrality, NE: local efficiency | | | | | | |

Compared with the MDD patients without NSSI history, the MDD patients with NSSI history showed significant decreases network in cerebellum-centre network(1084 edges, 114 nodes), including important nodes in the cerebellum (Vermis_6 67 edges), including the left thalamus (62 edges), the left angular gyrus (56 edges), and the right olfactory cortex (46 edges).

| eTable 13. MDD without NSSI vs with NSSI | | |
| --- | --- | --- |
| node | node | number |
| 112 | Vermis_6 | 67 |
| 77 | left thalamus | 62 |
| 65 | left angular gyrus | 56 |
| 22 | right olfactory cortex | 46 |
| 99 | Cerebelum_6_L | 40 |
| 42 | right amygdala | 39 |
| 85 | left middle temporal gyrus | 39 |
| 94 | Cerebelum_Crus2_R | 39 |
| 11 | left inferior frontal gyrus, opercular part | 38 |
| 81 | left superior temporal gyrus | 38 |
| 33 | left median cingulate and paracingulate gyri | 37 |
| 45 | left cuneus | 37 |
| 13 | left inferior frontal gyrus, triangular part | 36 |
| 91 | Cerebelum_Crus1_L | 34 |
| 61 | left inferior parietal, but supramarginal and angular gyri | 33 |
| 73 | left lenticular nucleus, putamen | 32 |
| 97 | Cerebelum_4_5_L | 32 |
| 98 | Cerebelum_4_5_R | 32 |
| 46 | right cuneus | 31 |
| 103 | Cerebelum_8_L | 31 |
| 66 | right angular gyrus | 30 |
| 72 | right caudate nucleus | 28 |
| 93 | Cerebelum_Crus2_L | 28 |
| 104 | Cerebelum_8_R | 28 |
| 57 | left postcentral gyrus | 27 |
| 55 | left fusiform gyrus | 26 |
| 86 | right middle temporal gyrus | 26 |
| 89 | left inferior temporal gyrus | 26 |
| 102 | Cerebelum_7b_R | 26 |
| 113 | Vermis_7 | 26 |
| 41 | left amygdala | 25 |
| 53 | left inferior occipital gyrus | 25 |
| 63 | left supramarginal gyrus | 25 |
| 82 | right superior temporal gyrus | 24 |
| 83 | left temporal pole: superior temporal gyrus | 24 |
| 17 | left rolandic operculum | 23 |
| 78 | right thalamus | 23 |
| 92 | Cerebelum_Crus1_R | 23 |
| 44 | right calcarine fissure and surrounding cortex | 22 |
| 47 | left lingual gyrus | 22 |
| 67 | left precuneus | 22 |
| 51 | left middle occipital gyrus | 21 |
| 84 | right temporal pole: superior temporal gyrus | 21 |
| 95 | Cerebelum_3_L | 21 |
| 101 | Cerebelum_7b_L | 21 |
| 1 | left precental gyrus | 20 |
| 43 | left calcarine fissure and surrounding cortex | 20 |
| 49 | left superior occipital gyrus | 20 |
| 52 | right middle occipital gyrus | 20 |
| 59 | left superior parietal gyrus | 20 |
| 114 | Vermis_8 | 20 |
| 21 | left olfactory cortex | 19 |
| 37 | left hippocampus | 19 |
| 48 | right lingual gyrus | 18 |
| 108 | Cerebelum_10_R | 18 |
| 14 | right inferior frontal gyrus, triangular part | 17 |
| 100 | Cerebelum_6_R | 17 |
| 116 | Vermis_10 | 17 |
| 12 | right inferior frontal gyrus, opercular part | 16 |
| 2 | right precental gyrus | 15 |
| 7 | left middle frontal gyrus | 15 |
| 34 | right median cingulate and paracingulate gyri | 15 |
| 69 | left paracentral lobule | 15 |
| 110 | Vermis_3 | 15 |
| 15 | left inferior frontal gyrus, orbital part | 14 |
| 19 | left supplementary motor area | 14 |
| 3 | left superior frontal gyrus, dorsolateral | 13 |
| 8 | right middle frontal gyrus | 13 |
| 62 | right inferior parietal, but supramarginal and angular gyri | 13 |
| 90 | right inferior temporal gyrus | 13 |
| 106 | Cerebelum_9_R | 13 |
| 111 | Vermis_4_5 | 13 |
| 4 | right superior frontal gyrus, dorsolateral | 12 |
| 24 | right superior frontal gyrus, medial | 12 |
| 32 | right anterior cingulate and paracingulate gyri | 12 |
| 36 | right posterior cingulate gyrus | 12 |
| 18 | right rolandic operculum | 11 |
| 40 | right parahippocampal gyrus | 11 |
| 58 | right postcentral gyrus | 11 |
| 115 | Vermis_9 | 11 |
| 16 | right inferior frontal gyrus, orbital part | 10 |
| 29 | left insula | 10 |
| 50 | right superior occipital gyrus | 10 |
| 68 | right precuneus | 10 |
| 75 | left lenticular nucleus, pallidum | 10 |
| 76 | right lenticular nucleus, pallidum | 10 |
| 20 | right supplementary motor area | 9 |
| 23 | left superior frontal gyrus, medial | 9 |
| 39 | left parahippocampal gyrus | 9 |
| 54 | right inferior occipital gyrus | 9 |
| 56 | right fusiform gyrus | 9 |
| 105 | Cerebelum_9_L | 9 |
| 9 | left middle frontal gyrus, orbital part | 8 |
| 70 | right paracentral lobule | 8 |
| 71 | left caudate nucleus | 8 |
| 74 | right lenticular nucleus, putamen | 8 |
| 79 | left heschl gyrus | 8 |
| 31 | left anterior cingulate and paracingulate gyri | 7 |
| 87 | left temporal pole: middle temporal gyrus | 7 |
| 30 | right insula | 6 |
| 38 | right hippocampus | 6 |
| 64 | right supramarginal gyrus | 6 |
| 10 | right middle frontal gyrus, orbital part | 5 |
| 88 | right temporal pole: middle temporal gyrus | 5 |
| 28 | right gyrus rectus | 4 |
| 80 | right heschl gyrus | 4 |
| 6 | right superior frontal gyrus, orbital part | 3 |
| 25 | left superior frontal gyrus, medial orbital | 3 |
| 35 | left posterior cingulate gyrus | 3 |
| 60 | right superior parietal gyrus | 3 |
| 26 | right superior frontal gyrus, medial orbital | 2 |
| 27 | left gyrus rectus | 2 |
| 5 | left superior frontal gyrus, orbital part | 1 |
| 109 | Vermis_1_2 | 1 |
